# Cardiovascular events following coronavirus disease 2019 vaccination in adults: a nationwide Swedish study

**DOI:** 10.1093/eurheartj/ehae639

**Published:** 2024-09-30

**Authors:** Yiyi Xu, Huiqi Li, Ailiana Santosa, Björn Wettermark, Tove Fall, Jonas Björk, Mats Börjesson, Magnus Gisslén, Fredrik Nyberg

**Affiliations:** School of Public Health and Community Medicine, Institute of Medicine, Sahlgrenska Academy, University of Gothenburg, BOX 414, 40 530 Gothenburg, Sweden; School of Public Health and Community Medicine, Institute of Medicine, Sahlgrenska Academy, University of Gothenburg, BOX 414, 40 530 Gothenburg, Sweden; School of Public Health and Community Medicine, Institute of Medicine, Sahlgrenska Academy, University of Gothenburg, BOX 414, 40 530 Gothenburg, Sweden; Pharmacoepidemiology and Social Pharmacy, Department of Pharmacy, Uppsala University, Uppsala, Sweden; Pharmacy Centre, Faculty of Medicine, Vilnius University, Vilnius, Lithuania; Department of Medical Sciences, Molecular Epidemiology and Science for Life Laboratory, Uppsala University, Uppsala, Sweden; Department of Laboratory Medicine, Division of Occupational and Environmental Medicine, Lund University, Lund, Sweden; Clinical Studies Sweden, Forum South, Skåne University Hospital, Lund, Sweden; Department of Molecular and Clinical Medicine, Institute of Medicine, Sahlgrenska Academy, University of Gothenburg, Gothenburg, Sweden; Centre for Lifestyle Intervention, Sahlgrenska University Hospital, Region Västra Götaland, Gothenburg, Sweden; Department of Infectious Diseases, Institute of Biomedicine, Sahlgrenska Academy, University of Gothenburg, Gothenburg, Sweden; Department of Infectious Diseases, Sahlgrenska University Hospital, Region Västra Götaland, Gothenburg, Sweden; Public Health Agency of Sweden, Solna, Sweden; School of Public Health and Community Medicine, Institute of Medicine, Sahlgrenska Academy, University of Gothenburg, BOX 414, 40 530 Gothenburg, Sweden

**Keywords:** COVID-19 vaccine, Cardiovascular diseases, Survival analysis

## Abstract

**Background and Aims:**

While the rationale for coronavirus disease 2019 (COVID-19) vaccination is to reduce complications and overall mortality, some cardiovascular complications from the vaccine itself have been demonstrated. Myocarditis and pericarditis are recognized as rare acute adverse events after mRNA vaccines in young males, while evidence regarding other cardiovascular events remains limited and inconsistent. This study assessed the risks of several cardiovascular and cerebrovascular events in a Swedish nationwide register-based cohort.

**Methods:**

Post-vaccination risk of myocarditis/pericarditis, dysrhythmias, heart failure, myocardial infarction, and cerebrovascular events (transient ischaemic attack and stroke) in several risk windows after each vaccine dose were assessed among all Swedish adults (*n* = 8 070 674). Hazard ratios (HRs) with 95% confidence intervals (95% CIs) compared with unvaccinated were estimated from Cox regression models adjusted for potential confounders.

**Results:**

For most studied outcomes, decreased risks of cardiovascular events post-vaccination were observed, especially after dose three (HRs for dose three ranging from .69 to .81), while replicating the increased risk of myocarditis and pericarditis 1–2 weeks after COVID-19 mRNA vaccination. Slightly increased risks, similar across vaccines, were observed for extrasystoles [HR 1.17 (95% CI 1.06–1.28) for dose one and HR 1.22 (95% CI 1.10–1.36) for dose two, stronger in elderly and males] but not for arrhythmias and for transient ischaemic attack [HR 1.13 (95% CI 1.05–1.23), mainly in elderly] but not for stroke.

**Conclusions:**

Risk of myopericarditis (mRNA vaccines only), extrasystoles, and transient ischaemic attack was transiently increased after COVID-19 vaccination, but full vaccination substantially reduced the risk of several more severe COVID-19-associated cardiovascular outcomes, underscoring the protective benefits of complete vaccination.

## Structured Graphical Abstract

**Structured Graphical Abstract:**
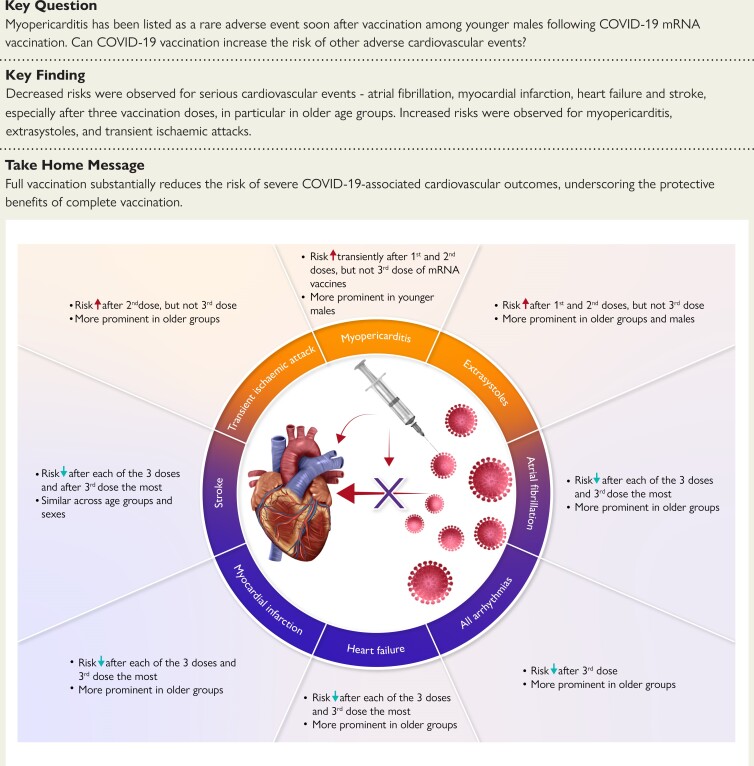
Conceptual framework and key results from a study of cardiovascular events following COVID-19 vaccination, based on a nationwide cohort of 8 million Swedish adults followed from December 2020 to December 2022.

**See the editorial comment for this article ‘Overall cardioprotective effects of COVID mRNA vaccines', by C.R. MacIntyre, https://doi.org/10.1093/eurheartj/ehae800.**

## Introduction

The rationale for recommending coronavirus disease 2019 (COVID-19) vaccination is to reduce infection risk, complications, and case fatality. However, the vaccination itself may also increase the risks of some cardiovascular events. Myocarditis and pericarditis have been listed by the European Medicines Agency as very rare (<1/10 000) adverse events after mRNA vaccine towards severe acute respiratory syndrome-coronavirus 2 (SARS-CoV-2), with the highest incidence observed in young males, primarily within 14 days, and potentially more common after the second dose.^1,2^ Supporting evidence has been reported from the USA, Israel, England, Korea, and four Nordic countries.^3–8^ These findings raise the question if other cardiovascular health outcomes could also be affected.

Early data from clinical trials by Pfizer and Moderna indicated a low incidence of myocardial infarction (MI) in vaccinated individuals.^9,10^ Case reports have described acute MI events shortly after vaccination, especially in the elderly,^11,12^ but supporting evidence from population-based studies is largely lacking. One case series study with self-control in France found no increased incidence of acute MI within 14 days after the Pfizer mRNA vaccine among people ≥ 75 years.^13^ In a Korean cohort study, full prior vaccination among adults ≥ 18 years was associated with reduced risk of MI in 31–120 days after COVID-19 diagnosis.^14^ Despite isolated reports of MI occurring after vaccination, the current body of evidence does not support that vaccination entails an increased risk of MI. Presumably, this could relate to vaccination protecting against severe COVID-19 infection, with consequent reduced risk of MI—a known cardiovascular complication of COVID-19.^15,16^

Similarly, case reports have been published for atrial fibrillation (AF) after vaccination.^17^ United States studies using the Vaccine Adverse Event Reporting System (VAERS) data^18,19^ and a population-based UK study^5^ also provided evidence of a possible correlation between mRNA vaccination and AF. However, the incidence of AF was overall very low.

A recent review summarizing case reports on stroke after vaccination found that most cases were women under 60, however, with unclear incidence.^20^ One study using disproportionality analysis showed a potential signal for ischaemic stroke and transient ischaemic attack (TIA) after some mRNA vaccines and the AZD1222 vaccine,^21^ while the French and Korean population-based studies mentioned, with individual-level data, found no evidence of increased risk of stroke after vaccination; rather the risk was reduced.^13,14^

The evidence from population-based studies regarding cardiovascular adverse events other than myocarditis and pericarditis thus remains limited and inconsistent. We hypothesized that a mechanism that would generate an increased risk of myocarditis and pericarditis in younger age groups, particularly men, could manifest as other adverse cardiovascular events related to inflammation, cardiac rhythm disruptions, heart failure (HF), or arterial thromboembolic events, in other age groups and potentially with different time lags. Therefore, in this nationwide register-based cohort study including all Swedish adults, we assessed the risks of several cardiovascular and cerebrovascular events—all within a range of risk windows after one, two, and three doses.

## Methods

### Study design, data source, and study population

This nationwide register-based cohort study was conducted as part of the RECOVAC (register-based large-scale national population study to monitor COVID-19 vaccination effectiveness and safety) study effort within the SCIFI-PEARL (Swedish COVID-19 Investigation for Future Insights—a Population Epidemiology Approach using Register Linkage) project.^22^ In the current study, we included all adults in Sweden born before 2003 (aged ≥18 in 2021), who were alive and residing in Sweden on 1 January 2018 (to ensure a minimum lookback time for pre-index covariates) and still resident on 27 December 2020 (the study index date), resulting in 8 070 674 individuals. Detailed data on socio-demographic, medical history, and COVID-19 vaccination were obtained through linkage with Swedish national and regional registers (see Supplementary data online, *Method*).

### Study period, exposure, and risk windows

The exposure variables were each dose of any COVID-19 vaccine from 27 December 2020 (start date of vaccinations in Sweden) to 31 December 2022. An individual’s exposure status was ‘unvaccinated’ until dose one was given, and then, his/her exposure status was changed to ‘dose one’. Subsequently, the exposure status was changed to dose two once dose two was given, and so on. Within each dose, five mutually exclusive risk windows of interest [1–7 days (Week 1), 8–14 days (Week 2), 15–21 days (Week 3), 22–28 days (Week 4), and 29–42 days (Week 5–6)] were defined. After Week 6, individuals were classified as exposed for that dose but outside the risk windows of interest, until they received the next dose. Individuals who received the next dose before the end of the fifth risk window of a particular dose would only contribute to the risk windows up until that date for the previous dose.

### Outcomes

The cardiovascular outcomes were defined using the Swedish clinical modification of the International Classification of Diseases, 10th revision (ICD-10-SE) as listed in *Table 1*. They were myocarditis/pericarditis (largely as ‘positive control’); dysrhythmias, including extrasystoles, AF, and arrhythmias overall; cardiac conditions, including MI and HF; and cerebrovascular events including TIA and stroke (both ischaemic and haemorrhagic). We also analysed the combined outcomes of first myocarditis or pericarditis (‘myopericarditis’) and the composite of TIA or stroke (since TIA may be considered a less serious version of stroke) to increase statistical power.

**Table 1:** ICD-10-SE codes used for each cardiovascular outcome

Cardiovascular outcomes	ICD-10-SE codes
Myocarditis	I012, I090, I40, I41, I514
Pericarditis	I010, I092, I30, I310, I311, I32
Extrasystoles	I491, I492, I493, I494
Atrial fibrillation	I48
Arrhythmias, all	I44, I45, I46, I47, I48, I49, R000, R001
Myocardial infarction	I21
Heart failure	I50
Transient ischaemic attack	G45
Ischaemic stroke	I63
Haemorrhagic stroke	I61, I62
Stroke, all	I61, I62, I63, I64

ICD-10-SE, Swedish clinical modification of the International Statistical Classification of Diseases and Related Health Problems, 10th revision (https://www.socialstyrelsen.se/statistik-och-data/klassifikationer-och-koder/icd-10/).

Each outcome was defined as incident events using the first ICD-coded primary or secondary diagnosis from the National Patient Register (including both specialist outpatient visits and hospital admissions) or underlying or contributing cause of death in the Cause of Death Register, during the study period. For each outcome, individuals with any prior registered record from 1 January 2015 to 26 December 2020 of that particular diagnosis were excluded.

### Covariates

A list of potential confounders was pre-determined after literature review, and a directed acyclic graph was adapted to select covariates into the statistical model. The selected covariates included age (cubic spline with four knots) and COVID-19 infection (yes/no) as time-varying covariates (see below) and baseline sex (male, female), country of birth (Swedish-born/foreign-born), employed as a healthcare worker (yes/no), marital status (married/not married), education (primary/secondary/tertiary/unknown), health-seeking behaviours (assessed by number of specialist outpatient visits, and days of inpatient stay, during 2018–19), as well as relevant prior comorbidities and treatments (yes/no) (see Supplementary data online, *Table S1*). Health-seeking behaviours and prior comorbidities were considered surrogates for baseline health status and access to care.

Baseline covariates were defined on or before 1 January 2020. Age was time varying as it changes for each calendar year. Coronavirus disease 2019 infection was also time varying and coded as ‘no’ until the first positive SARS-CoV-2 PCR test was identified from SmiNet, the national register of notifiable communicable diseases.

### Statistical analyses

Cox proportional hazard models with time-varying exposure were used, with calendar time as the underlying time scale. This means that risk sets were assessed sequentially in the Cox analysis each day, thus by design controlling for calendar time-related factors such as infection pressure or pandemic restrictions. Everyone’s follow-up time was first divided according to his/her vaccine status (unvaccinated, first dose, second dose, third dose) and then by the weekly risk windows within each dose. Each individual was followed from the index date until the earliest of the outcome of interest, end of each risk window, or a censoring event (fourth dose of any COVID-19 vaccine, emigration, death, or 31 December 2022). An individual contributed person-time as unvaccinated until the first vaccination. After each dose, individuals contributed person-time in each corresponding risk window of interest (i.e. exposed risk-time).

We first performed analyses for any vaccine product regardless of risk windows and estimated the overall hazard ratio (HR) for cardiovascular outcomes within 42 days (Weeks 1–6) after each vaccination dose compared with unvaccinated person-time. Then, we estimated HRs for any vaccine product after each dose for each risk window. Three stratified analyses were performed based on age, sex, and three vaccine products mainly used in Sweden: BNT162b2 (Pfizer-BioNTech), mRNA-1273 (Moderna), and AZD1222 (AstraZeneca). Age-stratified analyses used five pre-determined age groups, 18–25, 26–40, 41–65, 66–75, and ≥76 years (for some outcomes, some age groups were omitted due to few events). Sex-stratified analyses used an appropriate combination of the five age groups, for different outcomes, according to previous published evidence and number of available events in the study population. For myopericarditis, sex-stratified analysis was restricted to the age range 18–40 years, as they showed the largest elevated risks. For the other outcomes, sex-stratified analyses were restricted to the age range ≥ 41 years, as very few events occurred among young adults. Vaccine product-stratified analyses were also performed in the corresponding combined age categories. Additionally, in the vaccine product-stratified analyses, overall HRs were estimated for 28 days after administration of each dose due to limited numbers of events in weekly time windows. For myopericarditis, focusing on younger adults below 40 years, we only included two vaccine products (BNT162b2 and mRNA-1273) since AZD1222 was rarely used among young adults in Sweden, and we only estimated HRs for two doses as few individuals received a third dose. For other outcomes, dose three of AZD1222 was not included in the vaccine product-stratified analyses, since AZD1222 was not recommended to individuals younger than 65 years after 16 March 2021 and very few individuals of any age (*n* = 342) received a third dose of AZD1222. We report HR with 95% confidence intervals (CIs) from a crude model and a full model that adjusted for all covariates mentioned. All statistical analyses were performed using a standard software package (Stata, version. 18.0; StataCorp LLC).

## Results

Among the 8 070 674 adults included in the study, a large majority (88.5%) received at least one dose, 86.9% at least two, and 67.9% three or more doses of COVID-19 vaccine. There were 1 668 508 new cases of COVID-19 infection during the study period from 27 December 2020 to 31 December 2022, among which 672 279 (40.3%) occurred before getting the first dose of vaccination, 61 018 (3.7%) between the first and second dose, 573 145 (34.3%) between the second and third dose, and 362 066 (21.7%) after getting the third dose.

The demographic and medical history of the study population is shown in *Table 2*. Individuals who received more doses were slightly older. Hypertension was the most common medical condition (24.3%), followed by conditions requiring antidepressant prescription (11.9%).

**Table 2:** Demographics and medical history in the Swedish adult (≥18 years) general population, by vaccine status

	Whole study population	Vaccine status at the end of follow-up			
	At baseline (all were unvaccinated) (*n* = 8 070 674)	Unvaccinated (*n* = 930 555)	At least one dose (*n* = 7 140 119)	At least two doses (*n* = 7 012 574)	Three doses and more (*n* = 5 478 618)
Age, median (p25–p75)	48 (32–64)	37 (27–52)	49 (33–65)	50 (33–66)	54 (39–69)
**Sex**					
Male	4 031 019 (49.9)	515 084 (55.4)	3 515 935 (49.2)	3 446 481 (49.1)	2 603 995 (47.5)
Female	4 039 655 (50.1)	415 471 (44.6)	3 624 184 (50.8)	3 566 093 (50.9)	2 874 623 (52.5)
**Country of birth**					
Sweden	6 438 797 (79.8)	529 867 (56.9)	5 908 930 (82.8)	5 828 002 (83.1)	4 749 390 (86.7)
Outside Sweden	1 631 877 (20.2)	400 688 (43.1)	1 231 189 (17.2)	1 184 572 (16.9)	729 228 (13.3)
**Employed as a healthcare worker**					
Yes	1 739 403 (21.6)	194 995 (21.0)	1 544 408 (21.6)	1 514 402 (21.6)	1 129 707 (20.6)
**Marital status**					
Married	3 267 159 (40.5)	259 346 (27.9)	3 007 813 (42.1)	2 975 868 (42.4)	2 535 634 (46.3)
Not married	4 797 100 (59.4)	665 971 (71.6)	4 131 129 (57.9)	4 036 123 (57.6)	2 942 751 (53.7)
Unknown	6415 (.1)	5238 (.5)	1177 (0)	583 (0)	233 (0)
**Education**					
Primary	1 553 291 (19.2)	229 658 (24.7)	1 323 633 (18.5)	1 285 956 (18.3)	939 057 (17.1)
Secondary	3 443 823 (42.7)	408 904 (43.9)	3 034 919 (42.5)	2 980 435 (42.5)	2 284 455 (41.7)
Tertiary	2 928 942 (36.3)	235 557 (25.3)	2 693 385 (37.7)	2 662 971 (38)	2 204 521 (40.2)
Unknown	144 618 (1.8)	56 436 (6.1)	88 182 (1.2)	83 212 (1.2)	50 585 (.9)
Hypertension (diagnosis or medication)	1 956 940 (24.3)	104 534 (11.2)	1 852 406 (25.9)	1 835 177 (26.2)	1 683 608 (30.7)
Diabetes (types 1 and 2)	513 378 (6.4)	33 899 (3.6)	479 479 (6.3)	473 877 (6.8)	421 952 (7.7)
Chronic pulmonary disease	89 111 (1.1)	6653 (.7)	82 458 (1.2)	80 959 (1.2)	71 819 (1.3)
Asthma	158 549 (2.0)	13 455 (1.4)	145 094 (2.0)	142 533 (2.0)	115 957 (2.1)
Chronic kidney disease	158 434 (2.0)	13 927 (1.5)	144 507 (2.0)	141 794 (2.0)	120 124 (2.2)
Cancer	418 551 (5.2)	21 207 (2.3)	397 344 (5.6)	393 315 (5.6)	362 385 (6.6)
Autoimmune disease	195 606 (2.4)	13 118 (1.4)	182 488 (2.6)	180 206 (2.6)	160 002 (2.9)
Thyroid diseases	496 037 (6.1)	39 363 (4.2)	456 674 (6.4)	450 782 (6.4)	389 045 (7.1)
Coagulation disorders	40 903 (.5)	3520 (.4)	37 383 (.5)	36 748 (.5)	30 738 (.6)
Psychiatric disorders	339 853 (4.2)	51 735 (5.6)	288 118 (4.0)	279 129 (4.0)	200 673 (3.7)
Antidepressant prescription	963 499 (11.9)	82 246 (8.8)	881 253 (12.3)	865 185 (12.3)	699 521 (12.8)
Obesity	153 021 (1.9)	17 383 (1.9)	135 638 (1.9)	132 660 (1.9)	101 362 (1.9)
Numbers of specialist outpatient visits, median (p25–p75)	3 (1–6)	3 (1–6)	3 (1–6)	3 (1–6)	3 (1–6)
Days of inpatient stay, median (p25–p75)	0 (0–1)	0 (0–1)	0 (0–1)	0 (0–1)	0 (0–1)

All individuals were unvaccinated at baseline and can contribute to more than one vaccine status group depending on how many doses they received. Data are number (percentage), unless otherwise stated.

### Myocarditis and pericarditis

**Figure 1:**
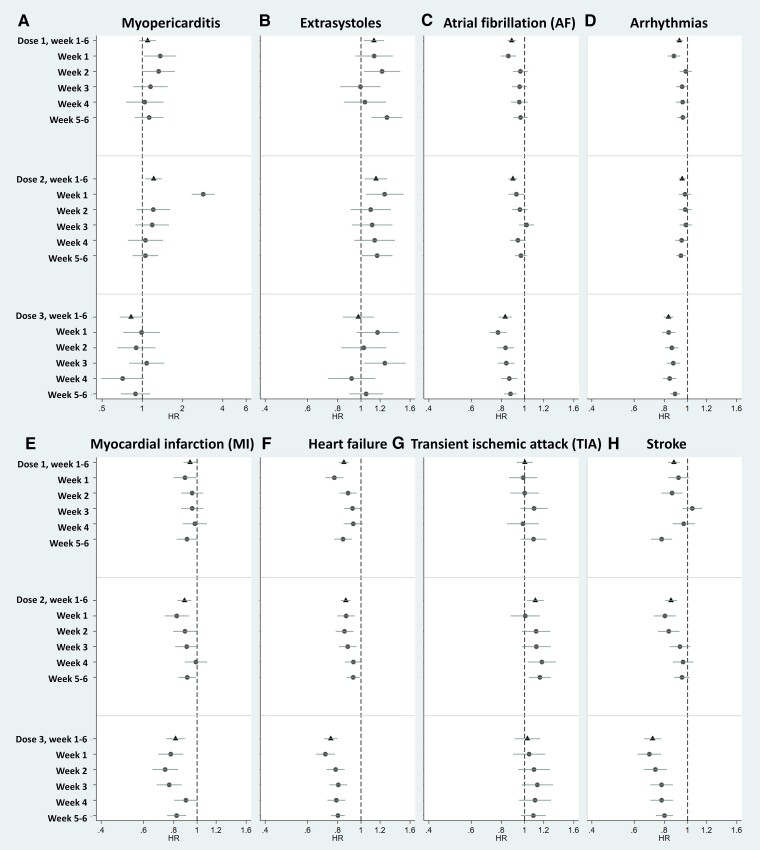
Forest plot showing hazard ratios for Weeks 1–6 (triangle) and for each weekly risk window (dot) with 95% confidence intervals for the investigated cardiovascular events. All events used composite endpoints, including specialist outpatient visits, hospital admissions, and deaths. The detailed data are presented in Supplementary data online, *Tables S2–S5*. Hazard ratio (triangle and dot) and 95% confidence intervals (lines) were obtained from a fully adjusted model. Note that the scales for hazard ratio are different between myopericarditis (range from .5 to 6) and the other cardiovascular events (range from .4 to 1.6)

An elevated early risk for myocarditis and pericarditis was observed after the first and second doses of vaccination. The effect size for myocarditis was larger, but the two outcomes showed similar dose and time window patterns (see Supplementary data online, *Table S2* and *Figure S1*) and were combined (myopericarditis) in the main analysis (*Figure 1A*; Supplementary data online, *Table S2*). The increased risk for myopericarditis started in the first week after dose one (HR 1.59, 95% CI 1.20–2.12) and remained up to the second week (HR 1.43, 1.06–1.94). The risk was higher in the first week after the second dose (HR 3.60, 2.94–4.40), but no effect for the third dose. The risk estimate was higher in ages 18–25 and 26–40 and in males (see Supplementary data online, *Figure S2*) and was present for both mRNA vaccines but was more prominent for mRNA-1273 than for BNT162b2 (*Table 3*).

**Table 3:** Hazard ratios with 95% confidence interval for cardiovascular events after vaccination with specific vaccine products after each dose within 28 days

Vaccine status	Vaccine product	Person-years	Cases	Incidence rate^a^	Full model^b^ HR (95% CI)
**Myopericarditis (18–40 years)**					
Unvaccinated		2 284 714	530	23.2	Ref
Dose one	BNT162b2	140 296	46	32.8	1.30 (.96, 1.76)
	mRNA-1273	32 659	17	52.1	1.83 (1.16, 2.90)
Dose two	BNT162b2	142 627	80	56.1	1.82 (1.39, 2.37)
	mRNA-1273	29 531	54	182.9	4.52 (3.25, 6.30)
**Extrasystoles (≥41 years)**					
Unvaccinated		2 460 454	2287	93.0	Ref
Dose one	BNT162b2	268 591	318	118.4	1.16 (1.02, 1.31)
	mRNA-1273	38 783	36	92.8	1.07 (.81, 1.40)
	AZD1222	49 534	88	177.7	1.24 (1.02, 1.50)
Dose two	BNT162b2	277 917	304	109.4	1.07 (.94, 1.22)
	mRNA-1273	39 305	53	134.8	1.31 (1.03, 1.68)
	AZD1222	42 795	63	147.2	1.13 (.88, 1.46)
Dose three	BNT162b2	215 852	280	129.7	1.08 (.89, 1.31)
	mRNA-1273	99 172	95	95.8	.84 (.67, 1.07)
**Atrial fibrillation (≥41 years)**					
Unvaccinated		2 383 717	15 432	647.4	Ref
Dose one	BNT162b2	255 591	2202	861.5	.88 (.84, .92)
	mRNA-1273	36 990	287	775.9	.82 (.74, .91)
	AZD1222	46 608	564	1210.1	.84 (.78, .91)
Dose two	BNT162b2	263 854	2398	908.8	.88 (.84, .92)
	mRNA-1273	37 486	331	883.0	.90 (.82, .99)
	AZD1222	39 922	453	1134.7	.87 (.79, .95)
Dose three	BNT162b2	201 357	2330	1157.1	.85 (.79, .91)
	mRNA-1273	95 246	647	679.3	.80 (.73, .87)
**Arrhythmias (≥41 years)**					
Unvaccinated		2 339 293	23 559	1007.1	
Dose one	BNT162b2	249 695	3153	1262.7	.57 (.55, .59)
	mRNA-1273	36 155	421	1164.4	.55 (.53, .59)
	AZD1222	45 272	828	1829.0	.49 (.47, .52)
Dose two	BNT162b2	257 562	3396	1318.5	.58 (.56, .60)
	mRNA-1273	36 624	492	1343.4	.57 (.54, .60)
	AZD1222	38 687	634	1638.8	.49 (.47, .51)
Dose three	BNT162b2	195 201	3206	1642.4	.63 (.61, .65)
	mRNA-1273	93 208	959	1028.9	.60 (.58, .62)
**Myocardial infarction (≥41 years)**					
Unvaccinated		2 439 611	7972	326.8	Ref
Dose one	BNT162b2	265 677	1031	388.1	.89 (.84, .96)
	mRNA-1273	38 365	140	364.9	.85 (.73, .99)
	AZD1222	48 861	262	536.2	.86 (.77, .97)
Dose two	BNT162b2	274 788	1020	371.2	.87 (.81, .93)
	mRNA-1273	38 884	164	421.8	.99 (.86, 1.13)
	AZD1222	42 125	182	432.1	.74 (.64, .86)
Dose three	BNT162b2	212 667	993	466.9	.76 (.69, .84)
	mRNA-1273	98 225	315	320.7	.76 (.67, .85)
**Heart failure (≥41 years)**					
Unvaccinated		2 425 807	12 031	496.0	Ref
Dose one	BNT162b2	262 581	1790	681.7	.47 (.45, .50)
	mRNA-1273	37 917	225	593.4	.50 (.46, .54)
	AZD1222	48 543	319	657.1	.36 (.33, .38)
Dose two	BNT162b2	271 310	1908	703.3	.45 (.43, .48)
	mRNA-1273	38 430	240	624.5	.48 (.45, .52)
	AZD1222	41 810	274	655.3	.34 (.31, .36)
Dose three	BNT162b2	209 376	1940	926.6	.47 (.45, .49)
	mRNA-1273	97 393	527	541.1	.47 (.44, .49)
**Transit ischaemic attack (≥41 years)**					
Unvaccinated		2 449 641	4324	176.5	Ref
Dose one	BNT162b2	266 646	614	230.3	.96 (.88, 1.05)
	mRNA-1273	38 513	82	212.9	.99 (.82, 1.21)
	AZD1222	49 077	182	370.8	1.18 (1.03, 1.35)
Dose three	BNT162b2	275 794	674	244.4	1.07 (.98, 1.17)
	mRNA-1273	39 031	109	279.3	1.35 (1.15, 1.60)
	AZD1222	42 335	130	307.1	1.08 (.91, 1.29)
Dose three	BNT162b2	213 531	629	294.6	1.05 (.92, 1.21)
	mRNA-1273	98 585	188	190.7	.93 (.79, 1.10)
**Stroke (≥41 years)**					
Unvaccinated		2 438 860	8877	364.0	Ref
Dose one	BNT162b2	264 879	1269	479.1	.91 (.86, .97)
	mRNA-1273	38 318	155	404.5	.88 (.76, 1.01)
	AZD1222	48 976	247	504.3	.78 (.69, .87)
Dose two	BNT162b2	273 833	1268	463.1	.88 (.82, .93)
	mRNA-1273	38 833	157	404.3	.78 (.68, .89)
	AZD1222	42 243	227	537.4	.74 (.65, .84)
Dose three	BNT162b2	211 882	1190	561.6	.75 (.68, .82)
	mRNA-1273	98 128	303	308.8	.68 (.61, .76)

All events used composite endpoints, including specialist outpatient visits, hospital admissions, and deaths.

^a^Incidence rate was calculated as per 100 000 person-years.

^b^Full model included age, sex, country of birth, employed as a healthcare worker, marital status, education, COVID-19 infection, and health-seeking behaviours during 2018–19 (i.e. no. of primary care visits and number of specialist outpatient visits), and prior comorbidities and treatments are listed in Supplementary data online, *Table S1*.

### Dysrhythmia

Increased risk of extrasystoles was observed from 1.17 in the 1st dose (95% CI 1.06–1.28) to 1.22 (1.10–1.36) in the 2nd dose (*Figure 1B*; Supplementary data online, *Table S3*). This trend was more prominent in elderly and males, with no obvious distinct time window (see Supplementary data online, *Figure S3*). For AF (*Figure 1C*; Supplementary data online, *Table S3*) and for arrhythmias overall (*Figure 1D*; Supplementary data online, *Table S3*), risks tended to be attenuated, in particular after the third dose across all risk windows, and the risk estimate was higher in older age groups (see Supplementary data online, *Figures S4* and *S5A*–*E*). Both sexes showed similar risk patterns (see Supplementary data online, *Figures S4* and *S5F*–*G*); no difference was seen between vaccine products (*Table 3*).

### Cardiac outcomes

We observed decreased risk of MI and HF after COVID-19 vaccination (*Figure 1E* and *F*; Supplementary data online, *Table S4*), particularly after the third dose (for MI, HR .81, .74–.89; for HF, HR .73, .69–.78). The most noticeable reduction in risks was observed in the eldest groups (see Supplementary data online, *Figures S6* and *S7A*–*C*), and both sexes showed similar risk patterns (see Supplementary data online, *Figures S6* and *S7D* and *E*). No difference was seen between vaccine products (*Table 3*).

### Transient ischaemic attack and stroke

For TIA, an overall increased risk was observed after the second dose (HR 1.13, 1.05–1.23), and increased risks were more prominent during later risk windows (*Figure 1G*). Stratification by age showed that TIA rarely occurred after vaccination in age groups below 40. In older age groups, strength of association increased with age (see Supplementary data online, *Figure S8A*–*C*), similar across sex (see Supplementary data online, *Figure S8D*–*E*) and vaccine products (*Table 3*).

Conversely, for stroke, risks tended to be reduced, particularly after the third dose (HR .69, .63–.74; *Figure 1H*), similar across older age groups and sexes (see Supplementary data online, *Figure S9A*–*E*). No obvious difference between vaccine products was observed, though AZD1222 tended to show the lowest HRs (*Table 3*). Results for ischaemic and haemorrhagic stroke were similar (see Supplementary data online, *Figure S10*, *Table S6*). For the composite of TIA or stroke, the pattern of the risk was more like the stroke pattern, showing a decreased risk after the third dose (see Supplementary data online, *Figure S11*).

## Discussion

Our data clearly replicate prior evidence on myocarditis and pericarditis, consistent with a previous large Nordic study (including Swedish data as in this study), a UK study, and a Korean cohort study.^3,5,8^ These three studies showed an increased incidence rate ratio after the first and second doses of mRNA vaccine within 28 days. Our results further reveal that the elevated risk is more acute, i.e. within 14 days after the first dose and 7 days after the second dose. The consistency of the results for this positive control outcome validates our analysis approach of using time-varying Cox regression to capture risk change within short periods after vaccination, which is novel and different from prior studies.

For most of the severe cardiovascular conditions, i.e. AF, MI, HF, and stroke, protective effects were observed after vaccination, especially after the third dose (*Structured Graphical Abstract*). This is not in line with our initial hypothesis that the mechanism leading to increased risk of myocarditis and pericarditis may also manifest other adverse cardiovascular events. However, such decreased risks are likely related to the vaccination protection against COVID-19 infection and severe disease^23,24^ as it is established that COVID-19 infection increases the risk of heart diseases both acutely and long term.^16^ Decreased risks of adverse cardiovascular events, including MI and stroke after vaccination, have also been observed in a large US cohort study including more than 1.9 million adults^25^ and a Korean study with about .6 million participants.^14^ In the US study,^25^ full vaccination, equivalent to three doses in our study, had a lower HR than partial vaccination. The combined evidence, now further refined with our results, implies that COVID-19 vaccination is likely to provide important protection against severe cardiovascular conditions, and that the third dose may confer benefit beyond two-dose schedules. Since the main purpose of the current study was to understand time-varying adverse outcomes after vaccination, it is difficult to disentangle if the observed protective effects are mediated through an effect against COVID-19 infection and its secondary progression to severe cardiovascular disease, or if the vaccines themselves by other mechanisms can prevent secondary complications—or both. For this, further studies with other study designs are needed. The observed stronger protective effects after the third dose may also be affected by healthy/survivor bias. An individual would then be less likely to get the third dose if he/she had a cardiovascular event after the first or second dose; therefore, those receiving the third dose could then be generally healthier and have lower predisposition for cardiovascular events.

A slightly increased risk of TIA was observed mainly after the second dose and during later risk windows, despite the decreased risk for stroke. One speculation could be that people are more sensitized to new symptoms noticed after vaccination; thus, the increased risk can more likely be observed for more subjective or transient symptoms than harder, more objective outcomes. Similarly, a slightly increased risk of extrasystoles was observed after the first and second doses, despite the lower risk for arrhythmias overall. One study based on the World Health Organization database reported that tachycardia is one of the common adverse events observed with COVID-19 vaccines, but no data were available only for extrasystoles.^26^

It should be noted that for outcomes showing slightly increased risks (myopericarditis, TIA, and extrasystoles), the incidence rates were generally lower than for the other outcomes that showed decreased risks (AF, MI, HF, and stroke). Additionally, considering that TIA is an acute and possibly less serious version of ischaemic stroke (though clearly associated with adverse stroke) and extrasystoles are a transient and generally self-limiting cardiovascular condition, a potential slightly increased risk after vaccination (with relatively low incidence rates) should not overshadow the importance of improving vaccination rates, particularly not in view of the protective effects seen on more serious cardiovascular outcomes and in older age group—the high risk group for COVID-19. Additionally, studies have shown that the clinical condition of vaccine-associated myopericarditis is less severe than COVID-19 infection-associated myopericarditis, again emphasizing the importance of vaccination.^4,27,28^

Several studies indicated that the Omicron variants of SARS-CoV-2 are less pathogenic than Alpha or Delta variants.^29,30^ However, COVID-19 still occurs in every season, and a high proportion of infections in the general population lead to persistent infections, which may seed new outbreaks in the community.^31,32^ The symptoms and severity of COVID-19 infection are more dependent on the person’s immunity than they are on the variant. The persistent infections may also contribute to cases with post-COVID-19 conditions (PCC).^33^ Coronavirus disease 2019 vaccination has been shown as an effective protective measure, even for PCC,^34^ and is the best way to protect those who are at increased risk of becoming seriously ill.^24^ This is especially important for older people, a population that usually shows higher infection rates and more severe symptoms than other ages. As vaccine protection wanes over time,^24^ getting a refill dose regularly maintains good protection and has been recommended to all people aged 65 and over and to people in risk groups in Sweden and the USA.

The population-based cohort design with large study size, including all Swedish adults, and comprehensive follow-up data through national registers are the major strengths. The large sample size allowed us to assess shorter risk windows than previous studies. We estimated risks weekly instead of within a 28-day risk window after each vaccination as used previously.^3,5^ We also took particular care to address potential confounding with rich data from multiple linked registers with full national coverage. Potential calendar time-dependent confounding was controlled by design and by use of the Cox model with calendar time as the underlying time scale.^35^ Linkage to registers provided medical histories since 2015 as a surrogate of individuals’ overall pre-pandemic health condition and was used for model adjustments. Coronavirus disease 2019 infection was considered, by adjusting for this event in the model in a time-varying manner. Nonetheless, misclassification of infection is possible since it was based on a registered positive PCR test in SmiNet, which only captures persons who got tested. Although testing at suspected infection was mandated and very widespread during most of the study period, testing propensity has been associated with socio-economic and access to tests.^28^ Nonetheless, direct information on access to healthcare was not available, nor personal behaviour data such as smoking, physical activity, or personal views on vaccination. For most of the studied cardiovascular outcomes such as MI, HF, and stroke, the diagnoses are robust and the date of healthcare contact represents well the date of disease onset; however, for a mild condition, e.g. extrasystoles, a possible delay of healthcare contact after disease onset and a possible misclassification may be likely. For AF, a chronic condition that can recur after a long time free of symptoms, misclassification so that some cases may not be truly *de novo* incident during the study period is possible, even though we excluded individuals with prior AF records almost 6 years before the study start. Additionally, lack of diagnoses recorded in primary care is another limitation, for some conditions also captured there. However, if such delay or misclassification or missing data from primary care can be assumed to occur similarly during vaccinated and unvaccinated periods, the HR estimate would remain unbiased.

## Conclusions

In this unique, complete nationwide cohort, we found decreased risks of several serious cardiovascular outcomes after COVID-19 vaccination, likely related to the protection of vaccination against severe COVID-19. Increased risks were observed for two relatively mild cardiovascular conditions, i.e. extrasystoles and TIA. We confirmed previously reported increased risks of myocarditis and pericarditis shortly after COVID-19 mRNA vaccination.^3^ Overall, our results clearly underscore the protective benefits of complete vaccination, especially for elderlies.

## Supplementary Material

ehae639_Supplementary_Data

## Declarations

### Data Availability

This study used pseudonymized individual-level data from Swedish healthcare registers that are not publicly available according to Swedish legislation. The data can be obtained from the respective Swedish data holders on the basis of ethics approval for the research in question, subject to relevant legislation, processes, and data protection.

### Pre-registered Clinical Trial Number

None supplied.

## References

[1] EMA . Signal assessment report on Myocarditis, pericarditis with Tozinameran (COVID-19 mRNA vaccine (nucleoside-modified) - COMIRNATY). https://www.ema.europa.eu/en/documents/prac-recommendation/signal-assessment-report-myocarditis-pericarditis-tozinameran-covid-19-mrna-vaccine-nucleosidemodified-comirnaty_en.pdf (17 September 2024, date last accessed).

[2] Gao J , Feng L, Li Y, Lowe S, Guo Z, Bentley R, et al A systematic review and meta-analysis of the association between SARS-CoV-2 vaccination and myocarditis or pericarditis. Am J Prev Med 2023;64:275–84. 10.1016/j.amepre.2022.09.002 36266115 PMC9510095

[3] Karlstad Ø , Hovi P, Husby A, Härkänen T, Selmer RM, Pihlström N, et al SARS-CoV-2 vaccination and myocarditis in a Nordic cohort study of 23 million residents. JAMA Cardiol 2022;7:600–12. 10.1001/jamacardio.2022.0583 35442390 PMC9021987

[4] Oster ME , Shay DK, Su JR, Gee J, Creech CB, Broder KR, et al Myocarditis cases reported after mRNA-based COVID-19 vaccination in the US from December 2020 to August 2021. JAMA 2022;327:331–40. 10.1001/jama.2021.24110 35076665 PMC8790664

[5] Patone M , Mei XW, Handunnetthi L, Dixon S, Zaccardi F, Shankar-Hari M, et al Risks of myocarditis, pericarditis, and cardiac arrhythmias associated with COVID-19 vaccination or SARS-CoV-2 infection. Nat Med 2022;28:410–22. 10.1038/s41591-021-01630-0 34907393 PMC8863574

[6] Sun CLF , Jaffe E, Levi R. Increased emergency cardiovascular events among under-40 population in Israel during vaccine rollout and third COVID-19 wave. Sci Rep 2022;12:6978. 10.1038/s41598-022-10928-z 35484304 PMC9048615

[7] Hviid A , Nieminen TA, Pihlström N, Gunnes N, Dahl J, Karlstad Ø, et al Booster vaccination with SARS-CoV-2 mRNA vaccines and myocarditis in adolescents and young adults: a Nordic cohort study. Eur Heart J 2024;45:1327–35. 10.1093/eurheartj/ehae056 38365960

[8] Cho JY , Kim KH, Lee N, Cho SH, Kim SY, Kim EK, et al COVID-19 vaccination-related myocarditis: a Korean nationwide study. Eur Heart J 2023;44:2234–43. 10.1093/eurheartj/ehad339 37264895 PMC10290868

[9] FDA . FDA Briefing Document: Pfizer-BioNTech COVID-19 Vaccine (BNT162, PF-07302048). Vaccines and related biological products advisory committee briefing document (10 December 2020). https://www.fda.gov/media/144246/download (17 September 2024, date last accessed).

[10] FDA . FDA Briefing Document: Moderna COVID-19 Vaccine. Vaccines and related biological products advisory committee meeting (17 December 2020). https://www.fda.gov/media/144434/download (17 September 2024, date last accessed)..

[11] Baronti A , Gentile F, Manetti AC, Scatena A, Pellegrini S, Pucci A, et al Myocardial infarction following COVID-19 vaccine administration: *Post Hoc, Ergo Propter Hoc*? Viruses 2022;14:1644. 10.3390/v14081644 36016266 PMC9413746

[12] Khiali S , Rezagholizadeh A, Behzad H, Bannazadeh Baghi H, Entezari-Maleki T. Current evidence of COVID-19 vaccination-related cardiovascular events. Postgrad Med 2023;135:102–20. 10.1080/00325481.2022.2161249 36567602

[13] Jabagi MJ , Botton J, Bertrand M, Weill A, Farrington P, Zureik M, et al Myocardial infarction, stroke, and pulmonary embolism after BNT162b2 mRNA COVID-19 vaccine in people aged 75 years or older. JAMA 2022;327:80–2. 10.1001/jama.2021.21699 34807248 PMC8609457

[14] Kim YE , Huh K, Park YJ, Peck KR, Jung J. Association between vaccination and acute myocardial infarction and ischemic stroke after COVID-19 infection. JAMA 2022;328:887–9. 10.1001/jama.2022.12992 35867050 PMC9449799

[15] Zuin M , Rigatelli G, Battisti V, Costola G, Roncon L, Bilato C. Increased risk of acute myocardial infarction after COVID-19 recovery: a systematic review and meta-analysis. Int J Cardiol 2023;372:138–43. 10.1016/j.ijcard.2022.12.032 36535564 PMC9755219

[16] Xie Y , Xu E, Bowe B, Al-Aly Z. Long-term cardiovascular outcomes of COVID-19. Nat Med 2022;28:583–90. 10.1038/s41591-022-01689-3 35132265 PMC8938267

[17] Chen CY , Hsieh MT, Wei CT, Lin CW. Atrial fibrillation after mRNA-1273 SARS-CoV-2 vaccination: case report with literature review. Risk Manag Healthc Policy 2023;16:209–14. 10.2147/RMHP.S402007 36798620 PMC9926928

[18] Kattubadi A , Solorzano J, Feng K, Brar V, Dominic P. COVID-19 vaccines and atrial fibrillation risk: a pharmacovigilance analysis. J Am Coll Cardiol 2022;79:1838. 10.1016/S0735-1097(22)02829-7

[19] Kumar A , Shariff M, Bhat V, DeSimone C, Deshmukh A. Atrial fibrillation after vaccination for COVID-19: analysis of the vaccine adverse event reporting system. J Interv Card Electrophysiol 2022;65:1–2. 10.1007/s10840-022-01263-4 35674855 PMC9175153

[20] Kakovan M , Ghorbani Shirkouhi S, Zarei M, Andalib S. Stroke associated with COVID-19 vaccines. J Stroke Cerebrovasc Dis 2022;31:106440. 10.1016/j.jstrokecerebrovasdis.2022.106440 35339857 PMC8894799

[21] Sodhi M , Samii A, Etminan M. A comparative safety study of reported neurological adverse events with three COVID-19 vaccines. J Neurol 2022;269:2301–3. 10.1007/s00415-021-10919-6 34999959 PMC8742704

[22] Nyberg F , Franzén S, Lindh M, Vanfleteren L, Hammar N, Wettermark B, et al Swedish COVID-19 investigation for future insights—a population epidemiology approach using register linkage (SCIFI-PEARL). Clin Epidemiol 2021;13:649–59. 10.2147/CLEP.S312742 34354377 PMC8331198

[23] Nordström P , Ballin M, Nordström A. Risk of infection, hospitalisation, and death up to 9 months after a second dose of COVID-19 vaccine: a retrospective, total population cohort study in Sweden. Lancet 2022;399:814–23. 10.1016/S0140-6736(22)00089-7 35131043 PMC8816388

[24] Xu Y , Li H, Kirui B, Santosa A, Gisslén M, Leach S, et al Effectiveness of COVID-19 vaccines over 13 months covering the period of the emergence of the Omicron variant in the Swedish population. Vaccines (Basel) 2022;10:2074. 10.3390/vaccines10122074 36560484 PMC9782222

[25] Jiang J , Chan L, Kauffman J, Narula J, Charney AW, Oh W, et al Impact of vaccination on major adverse cardiovascular events in patients with COVID-19 infection. J Am Coll Cardiol 2023;81:928–30. 10.1016/j.jacc.2022.12.006 36813689 PMC9939951

[26] Jeet Kaur R , Dutta S, Charan J, Bhardwaj P, Tandon A, Yadav D, et al Cardiovascular adverse events reported from COVID-19 vaccines: a study based on WHO database. Int J Gen Med 2021;14:3909–27. 10.2147/IJGM.S324349 34349544 PMC8326931

[27] Hamedi KR , Loftus G, Traylor L, Goodwin R, Arce S. Comparison of COVID-19 vaccine-associated myocarditis and viral myocarditis pathology. Vaccines (Basel) 2023;11:362. 10.3390/vaccines11020362 36851240 PMC9967770

[28] Lai FTT , Chan EWW, Huang L, Cheung CL, Chui CSL, Li X, et al Prognosis of myocarditis developing after mRNA COVID-19 vaccination compared with viral myocarditis. J Am Coll Cardiol 2022;80:2255–65. 10.1016/j.jacc.2022.09.049 36480967 PMC9721305

[29] Thiruvengadam R , Rizvi ZA, Raghavan S, Murugesan DR, Gosain M, Dandotiya J, et al Clinical and experimental evidence suggest omicron variant of SARS-CoV-2 is inherently less pathogenic than delta variant independent of previous immunity. Eur J Med Res 2023;28:421. 10.1186/s40001-023-01373-3 37821945 PMC10566023

[30] Barh D , Tiwari S, Rodrigues Gomes LG, Ramalho Pinto CH, Andrade BS, Ahmad S, et al SARS-CoV-2 variants show a gradual declining pathogenicity and pro-inflammatory cytokine stimulation, an increasing antigenic and anti-inflammatory cytokine induction, and rising structural protein instability: a minimal number genome-based approach. Inflammation 2023;46:297–312. 10.1007/s10753-022-01734-w 36215001 PMC9549046

[31] Gonzalez-Reiche AS , Alshammary H, Schaefer S, Patel G, Polanco J, Carreño JM, et al Sequential intrahost evolution and onward transmission of SARS-CoV-2 variants. Nat Commun 2023;14:3235. 10.1038/s41467-023-38867-x 37270625 PMC10239218

[32] Ghafari M , Hall M, Golubchik T, Ayoubkhani D, House T, MacIntyre-Cockett G, et al Prevalence of persistent SARS-CoV-2 in a large community surveillance study. Nature 2024;626:1094–101. 10.1038/s41586-024-07029-4 38383783 PMC10901734

[33] Davis HE , McCorkell L, Vogel JM, Topol EJ. Long COVID: major findings, mechanisms and recommendations. Nat Rev Microbiol 2023;21:133–46. 10.1038/s41579-022-00846-2 36639608 PMC9839201

[34] Lundberg-Morris L , Leach S, Xu Y, Martikainen J, Santosa A, Gisslén M, et al COVID-19 vaccine effectiveness against post-COVID-19 condition among 589 722 individuals in Sweden: population based cohort study. BMJ 2023;383:e076990. 10.1136/bmj-2023-076990 37993131 PMC10666099

[35] Lund LC , Støvring H, Pottegård A, Andersen M, Hallas J. Cox regression using a calendar time scale was unbiased in simulations of COVID-19 vaccine effectiveness & safety. J Clin Epidemiol 2023;156:127–36. 10.1016/j.jclinepi.2023.02.012 36806733 PMC9933854

