# Supplementary material for: Cardiovascular events following coronavirus disease 2019 vaccination in adults: a nationwide Swedish study
Source: Eur Heart J. 2024 Sep 30;46(2):147–57. doi: 10.1093/eurheartj/ehae639 (PMC11704415; doi:10.1093/eurheartj/ehae639)
Supplement: ehae639_Supplementary_Data [file ehae639_supplementary_data.zip › 20240523--suppl. materials_PL.docx]

Supplemental Materials

Contents

[Cardiovascular events following COVID-19 vaccination in adults: a nationwide Swedish study 1](#_Toc167369290)

[Supplemental Method 3](#_Toc167369291)

[Data source 3](#_Toc167369292)

[Reference 3](#_Toc167369293)

[Supplemental Figures 4](#_Toc167369294)

[Figure S1. Forest plot showing hazard ratios (HR) for week 1-6 (triangle) and for each risk window (dot) with 95% confidence intervals (CI) for the **myocarditis (a) and pericarditis (b)**. 4](#_Toc167369295)

[Figure S2. Forest plot showing hazard ratios (HR) for week 1-6 (triangle) and for each risk window (dot) with 95% confidence intervals (CI) for the **myopericarditis in age groups (a - e) and in males and females aged 18-40 years (f and g)**, separately. 5](#_Toc167369296)

[Figure S3. Forest plot showing hazard ratios (HR) for week 1-6 (triangle) and for each risk window (dot) with 95% confidence intervals (CI) for the **extrasystoles in age groups (a - e) and in males and females aged ≥ 41 years (f and g),** separately. 6](#_Toc167369297)

[Figure S4. Forest plot showing hazard ratios (HR) for week 1-6 (triangle) and for each risk window (dot) with 95% confidence intervals (CI) for the **atrial fibrillation (AF) in age groups (a - e) and in males and females aged ≥ 41 years (f and g),** separately. 7](#_Toc167369298)

[Figure S5. Forest plot showing hazard ratios (HR) for week 1-6 (triangle) and for each risk window (dot) with 95% confidence intervals (CI) for the **arrhythmias overall in age groups (a - e) and in males and females aged ≥ 41 years (f and g),** separately. 8](#_Toc167369299)

[Figure S6. Forest plot showing hazard ratios (HR) for week 1-6 (triangle) and for each risk window (dot) with 95% confidence intervals (CI) for the **myocardial infarction (MI) in older age groups (a - c) and in males and females aged ≥ 41 years (d and e),** separately. 9](#_Toc167369300)

[Figure S7. Forest plot showing hazard ratios (HR) for week 1-6 (triangle) and for each risk window (dot) with 95% confidence intervals (CI) for the **heart failure (HF) in older age groups (a - c) and in males and females aged ≥ 41 years (d and e),** separately. 10](#_Toc167369301)

[Figure S8. Forest plot showing hazard ratios (HR) for week 1-6 (triangle) and for each risk window (dot) with 95% confidence intervals (CI) for the **transit ischemic attack (TIA) in older age groups (a - c) and in males and females aged ≥ 41 years (d and e),** separately. 11](#_Toc167369302)

[Figure S9. Forest plot showing hazard ratios (HR) for week 1-6 (triangle) and for each risk window (dot) with 95% confidence intervals (CI) for the **stroke in older age groups (a - c) and in males and females aged ≥ 41 years (d and e),** separately. 12](#_Toc167369303)

[Figure S10. Forest plot showing hazard ratios (HR) for week 1-6 (triangle) and for each risk window (dot) with 95% confidence intervals (CI) for the **ischemic stroke (a) and hemorrhagic stroke (b)**. 13](#_Toc167369304)

[Figure S11. Forest plot showing hazard ratios (HR) for week 1-6 (triangle) and for each risk window (dot) with 95% confidence intervals (CI) for the **transit ischemic attack (TIA, a) stroke (b) and the composite of TIA and stroke (c)**. 14](#_Toc167369305)

[Supplemental Tables 15](#_Toc167369306)

[Table S1. List of prior comorbidities and treatments, and corresponding ICD-10-SE and ATC codes. The comorbidities were considered from 1 January 2015 to the date of study start, while the prior treatments were considered from 1 January 2018 to the date of study start 15](#_Toc167369307)

[Table S2. Hazard ratios (HR) with 95% confidence interval (CI) for **myocarditis and pericarditis** after each dose in each risk windows, among Swedish adults. Myocarditis and pericarditis used composite endpoints, including specialist outpatient visits, hospital admissions and deaths. 16](#_Toc167369308)

[Table S3. Hazard ratios (HR) with 95% confidence interval (CI) for **dysrhythmia** after each dose in each risk windows, among Swedish adults. All dysrhythmia used composite endpoints, including specialist outpatient visits, hospital admissions and deaths. 19](#_Toc167369309)

[Table S4. Hazard ratios (HR) with 95% confidence interval (CI) for **cardiac outcomes** after each dose in each risk windows, among Swedish adults. All cardiac outcomes used composite endpoints, including specialist outpatient visits, hospital admissions and deaths. 22](#_Toc167369310)

[Table S5. Hazard ratios (HR) with 95% confidence interval (CI) for **transit ischemic attack (TIA) and stroke** after each dose in each risk windows, among Swedish adults. TIA and stroke used composite endpoints, including specialist outpatient visits, hospital admissions and deaths. 24](#_Toc167369311)

[Table S6. Hazard ratios (HR) with 95% confidence interval (CI) for **ischemic stroke and hemorrhagic stroke** after each dose in each risk windows, among Swedish adults. Both strokes used composite endpoints, including specialist outpatient visits, hospital admissions and deaths. 27](#_Toc167369312)

## Supplemental Method

### Data source

The large SCIFI-PEARL (Swedish COVID-19 Investigation for Future Insights – a Population Epidemiology Approach using Register Linkage) project is a nationwide register-based longitudinal project, which links data from Swedish national and regional registries and healthcare databases for each Swedish individual (1).

For the current study purposes, sociodemographic data, including country of birth, education, occupation, and income, were obtained from Statistics Sweden databases, while in- and outpatient specialist care data were obtained from the National Patient Register (NPR, 2). Data on dispensed prescriptions came from the National Prescribed Drug Register, and mortality data from the National Cause of Death Register (NCDR). All data were available since 2015, except for the prescription data, which were available since 2018. Positive SARS-CoV-2 PCR test was identified from SmiNet, the national register of notifiable communicable diseases (3). COVID-19 vaccination data, including date of vaccination, dose number and vaccine product, were obtained from the National Vaccination Register (NVR). The vaccination data were cleaned based on a predefined protocol to handle unlikely short intervals registered between administration of vaccines, as described elsewhere (4, 5).

### Reference

1. Nyberg F, Franzén S, Lindh M, et al. Swedish Covid-19 Investigation for Future Insights - A Population Epidemiology Approach Using Register Linkage (SCIFI-PEARL). Clin Epidemiol 2021;13:649–59.

2. Ludvigsson JF, Almqvist C, Bonamy A-KE, et al. Registers of the Swedish total population and their use in medical research. Eur J Epidemiol 2016;31(2):125–36.

3. Rolfhamre P, Janson A, Arneborn M, Ekdahl K. SmiNet-2: Description of an internet-based surveillance system for communicable diseases in Sweden. Euro Surveill 2006;11:15-6.

4. Ljung R, Xu Y, Sundström A, et al. Association between SARS-CoV-2 vaccination and healthcare contacts for menstrual disturbance and bleeding in women before and after menopause: nationwide, register based cohort study. BMJ 2023;381:e074778.

5. Xu Y, Li H, Kirui B, et al. Effectiveness of COVID-19 Vaccines over 13 Months Covering the Period of the Emergence of the Omicron Variant in the Swedish Population. Vaccines 2022;10(12):2074.

## Supplemental Figures


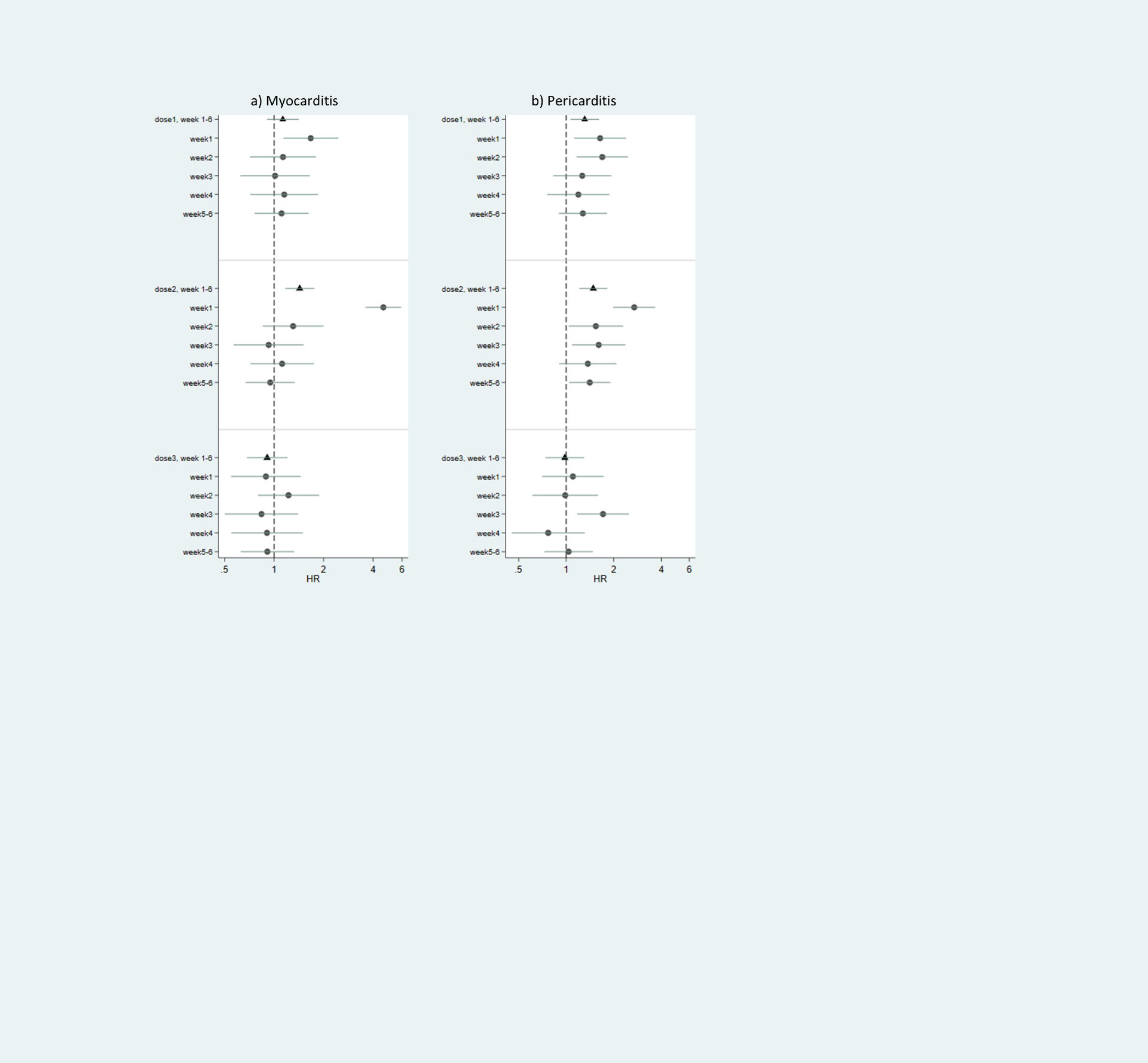


### Figure S1. Forest plot showing hazard ratios (HR) for week 1-6 (triangle) and for each risk window (dot) with 95% confidence intervals (CI) for the **myocarditis (a) and pericarditis (b)**.

The detailed data are presented in Supplemental Table S2. HR (triangle and dot) and 95%CI (lines) were obtained from a full adjusted model.

###
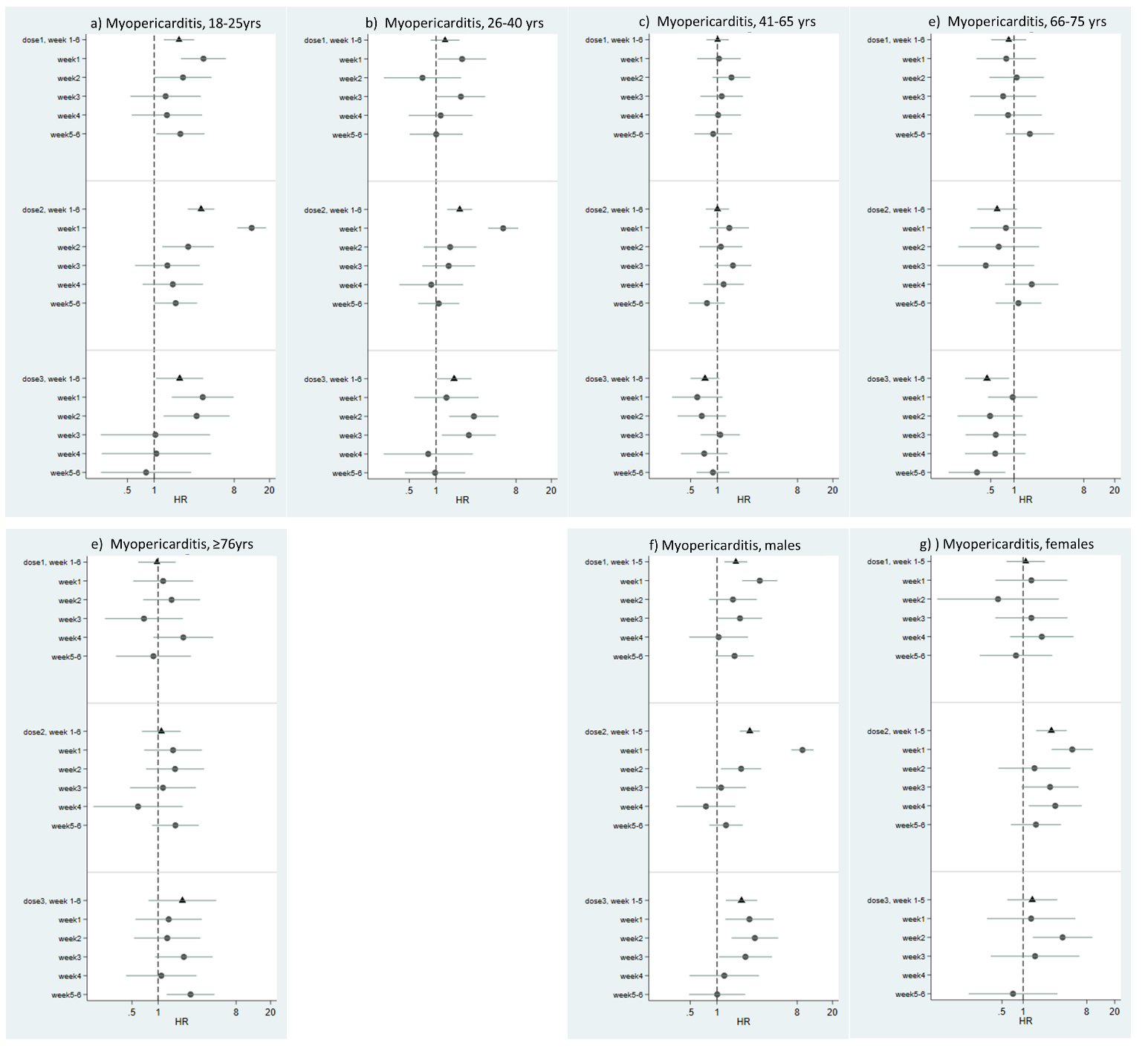
Figure S2. Forest plot showing hazard ratios (HR) for week 1-6 (triangle) and for each risk window (dot) with 95% confidence intervals (CI) for the **myopericarditis in age groups (a - e) and in males and females aged 18-40 years (f and g)**, separately.

HR (triangle and dot) and 95%CI (lines) were obtained from a full adjusted model.


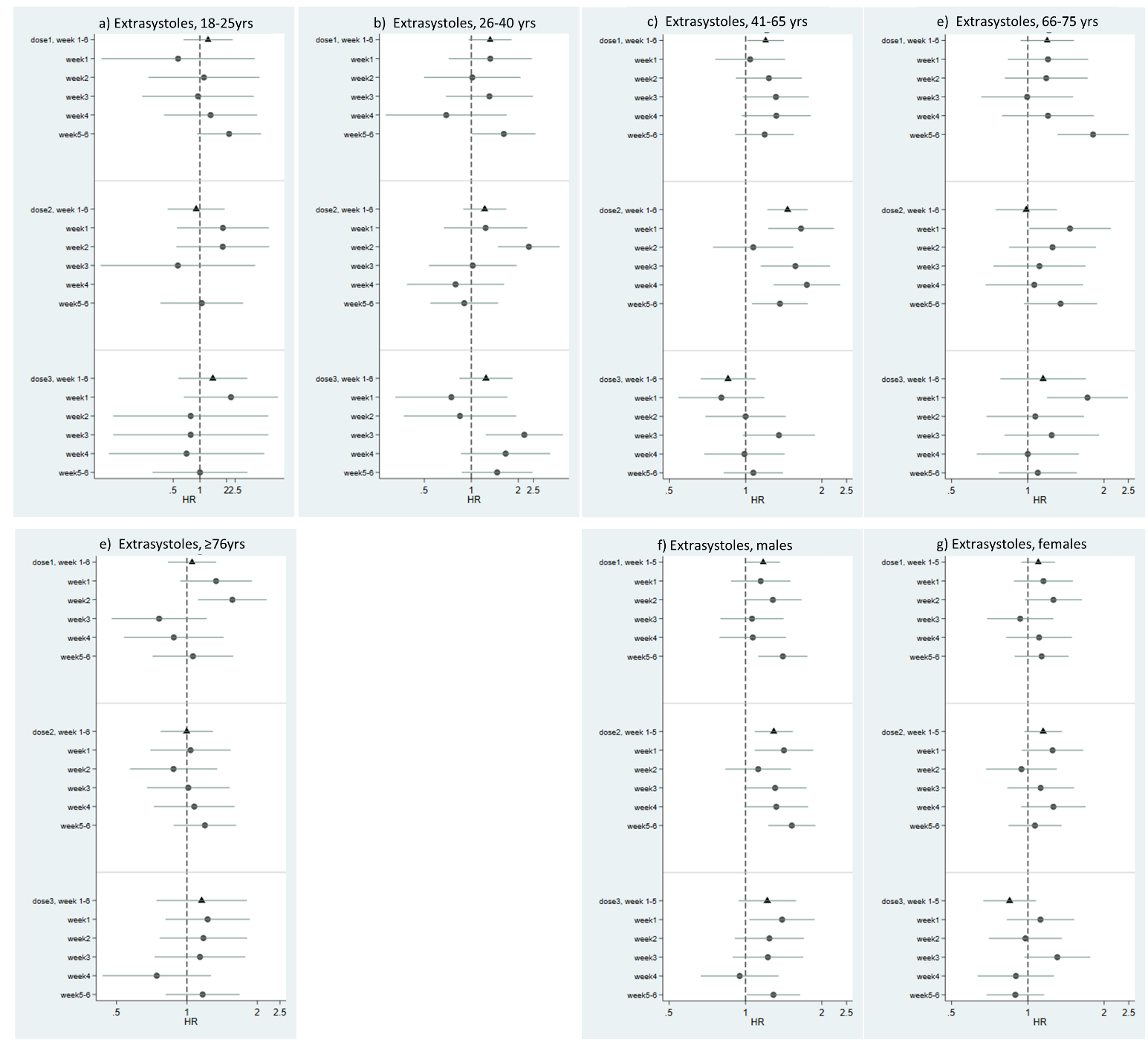


### Figure S3. Forest plot showing hazard ratios (HR) for week 1-6 (triangle) and for each risk window (dot) with 95% confidence intervals (CI) for the **extrasystoles in age groups (a - e) and in males and females aged ≥ 41 years (f and g),** separately.

HR (triangle and dot) and 95%CI (lines) were obtained from a full adjusted model.


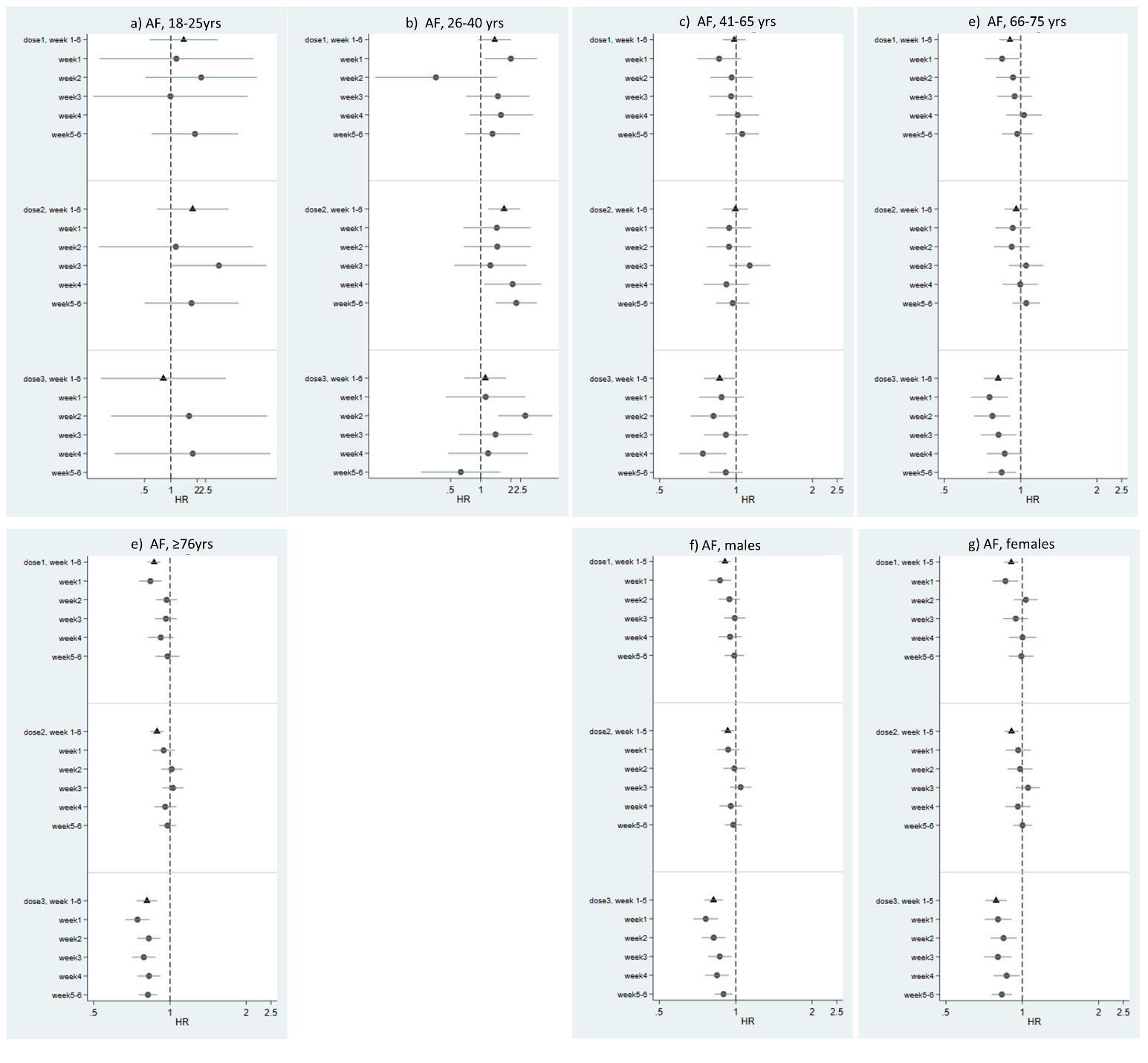


### Figure S4. Forest plot showing hazard ratios (HR) for week 1-6 (triangle) and for each risk window (dot) with 95% confidence intervals (CI) for the **atrial fibrillation (AF) in age groups (a - e) and in males and females aged ≥ 41 years (f and g),** separately.

HR (triangle and dot) and 95%CI (lines) were obtained from a full adjusted model.


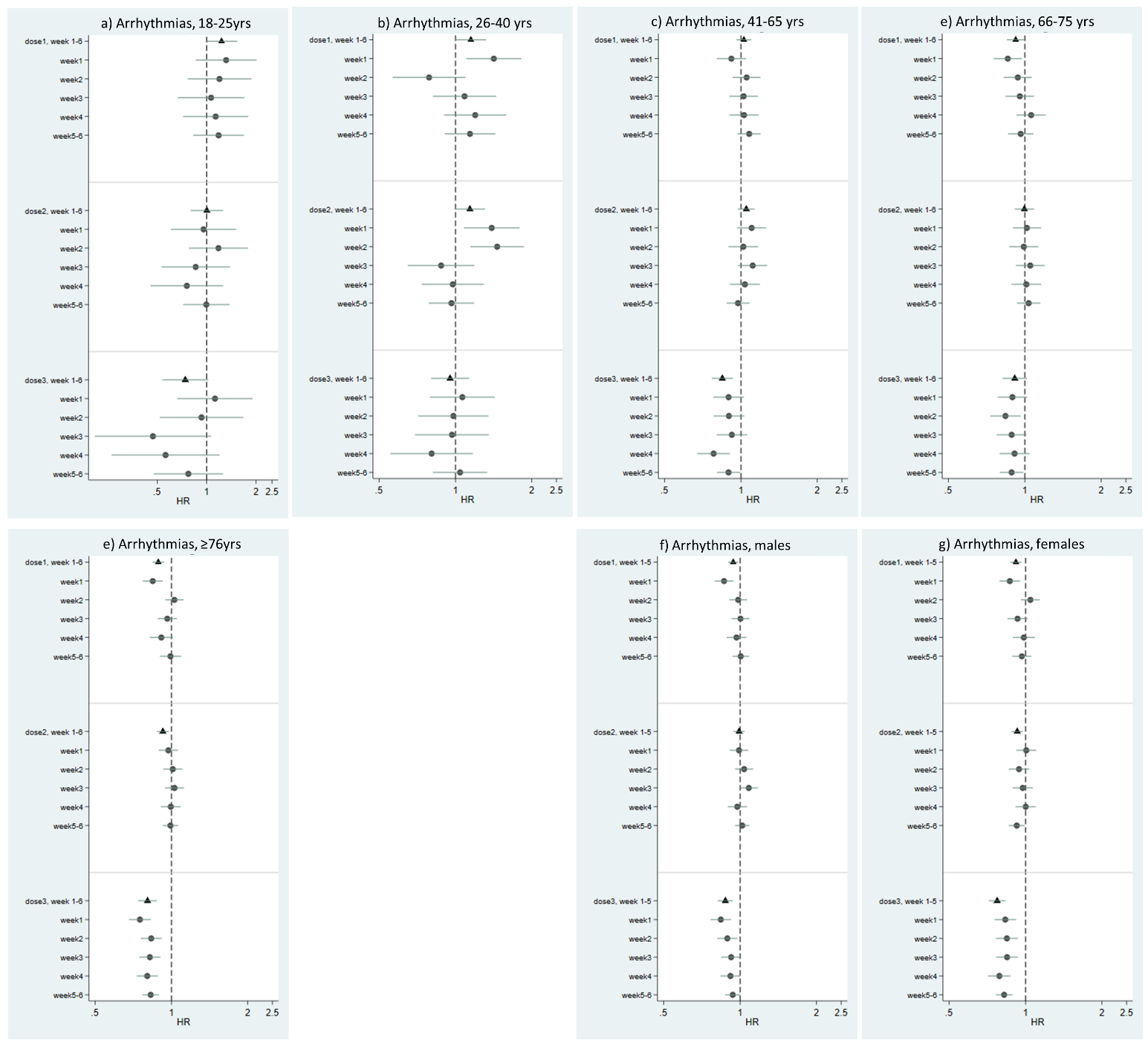


### Figure S5. Forest plot showing hazard ratios (HR) for week 1-6 (triangle) and for each risk window (dot) with 95% confidence intervals (CI) for the **arrhythmias overall in age groups (a - e) and in males and females aged ≥ 41 years (f and g),** separately.

HR (triangle and dot) and 95%CI (lines) were obtained from a full adjusted model.


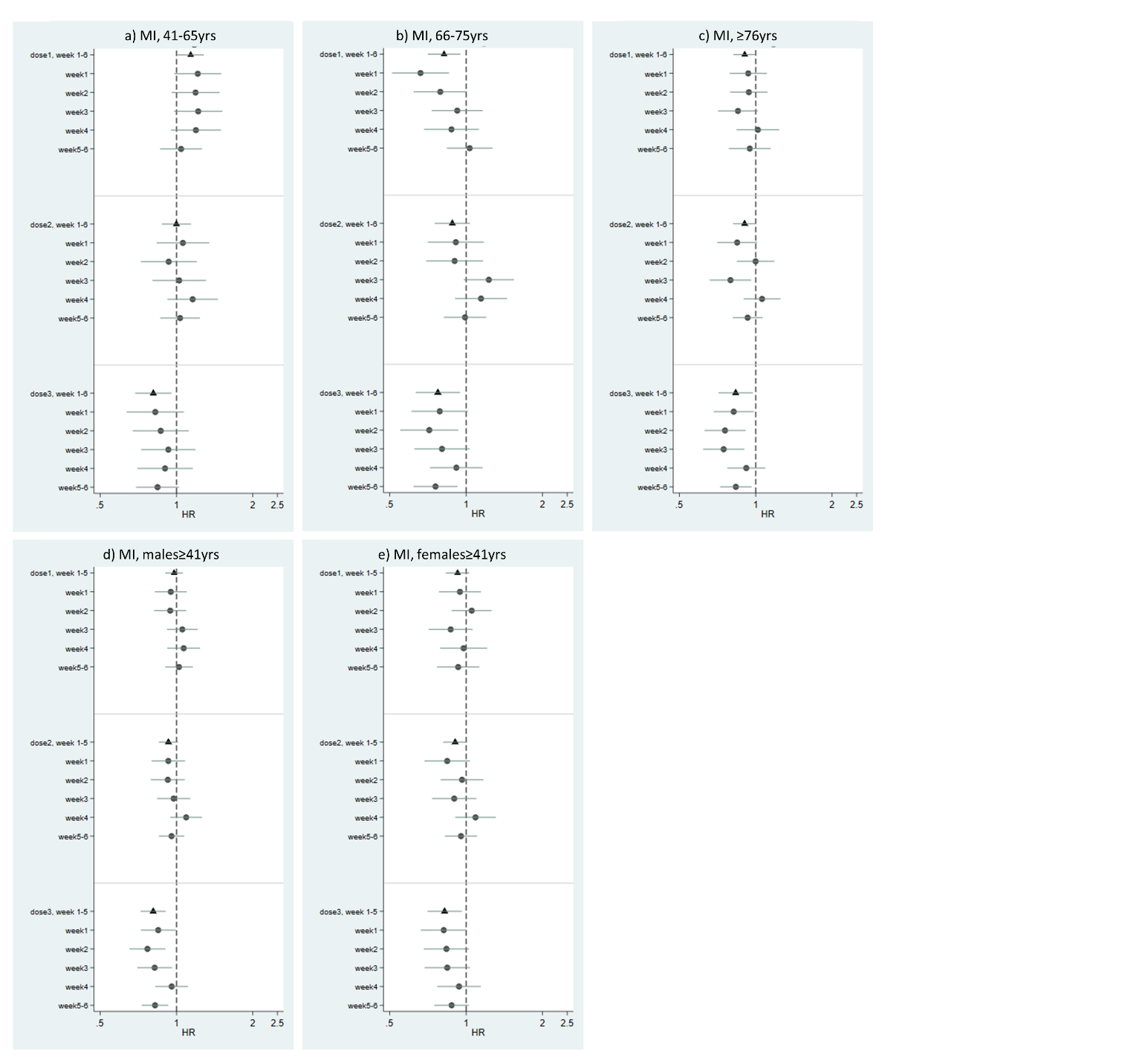


### Figure S6. Forest plot showing hazard ratios (HR) for week 1-6 (triangle) and for each risk window (dot) with 95% confidence intervals (CI) for the **myocardial infarction (MI) in older age groups (a - c) and in males and females aged ≥ 41 years (d and e),** separately.

HR (triangle and dot) and 95%CI (lines) were obtained from a full adjusted model. For age groups 18-25yrs and 26-40yrs, there were too few cases (<5 cases) to provide HR estimates for each risk window.


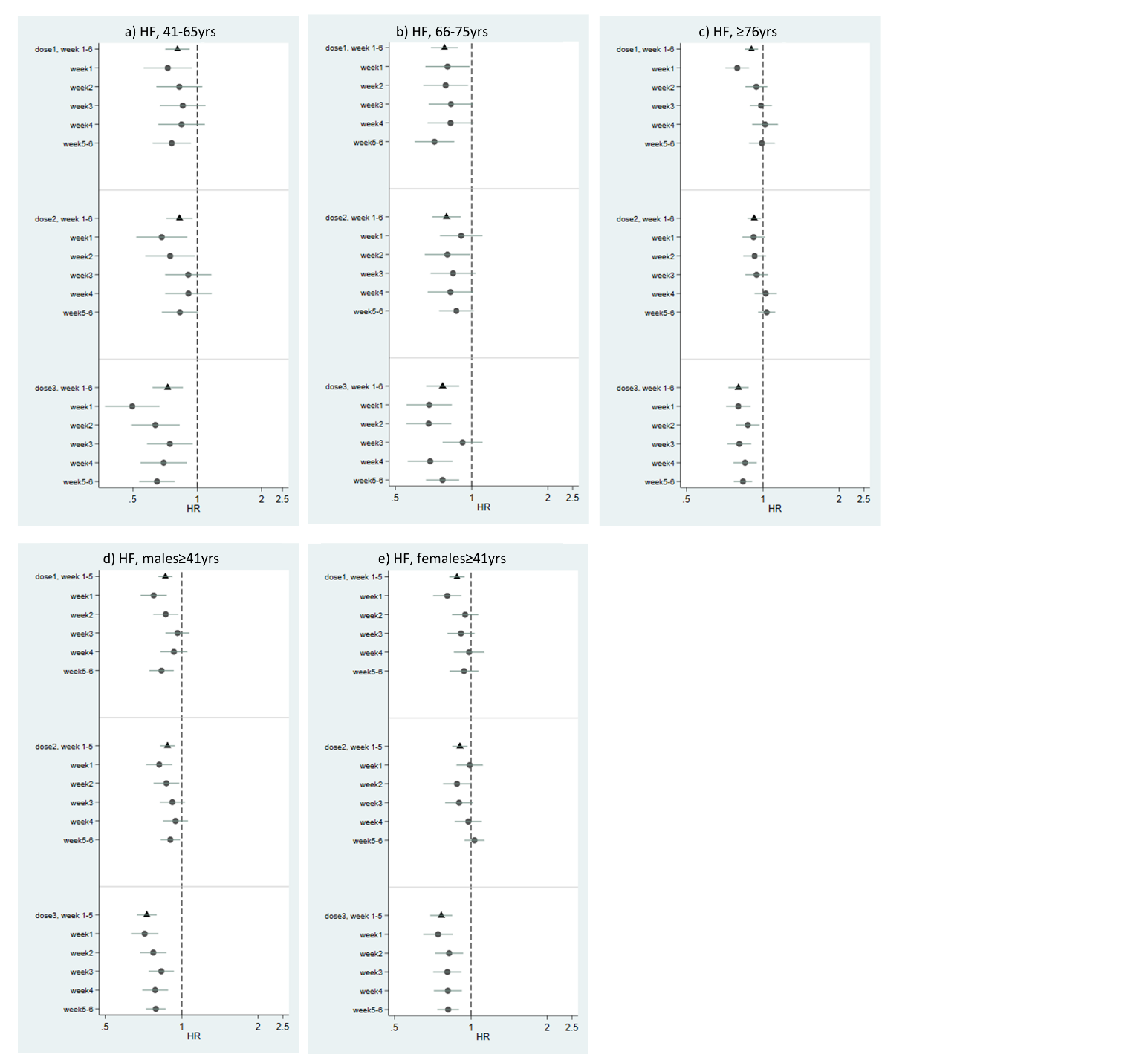


### Figure S7. Forest plot showing hazard ratios (HR) for week 1-6 (triangle) and for each risk window (dot) with 95% confidence intervals (CI) for the **heart failure (HF) in older age groups (a - c) and in males and females aged ≥ 41 years (d and e),** separately.

HR (triangle and dot) and 95%CI (lines) were obtained from a full adjusted model. For age groups 18-25yrs and 26-40yrs, there were too few cases (<5 cases) to provide HR estimates for each risk window.

###
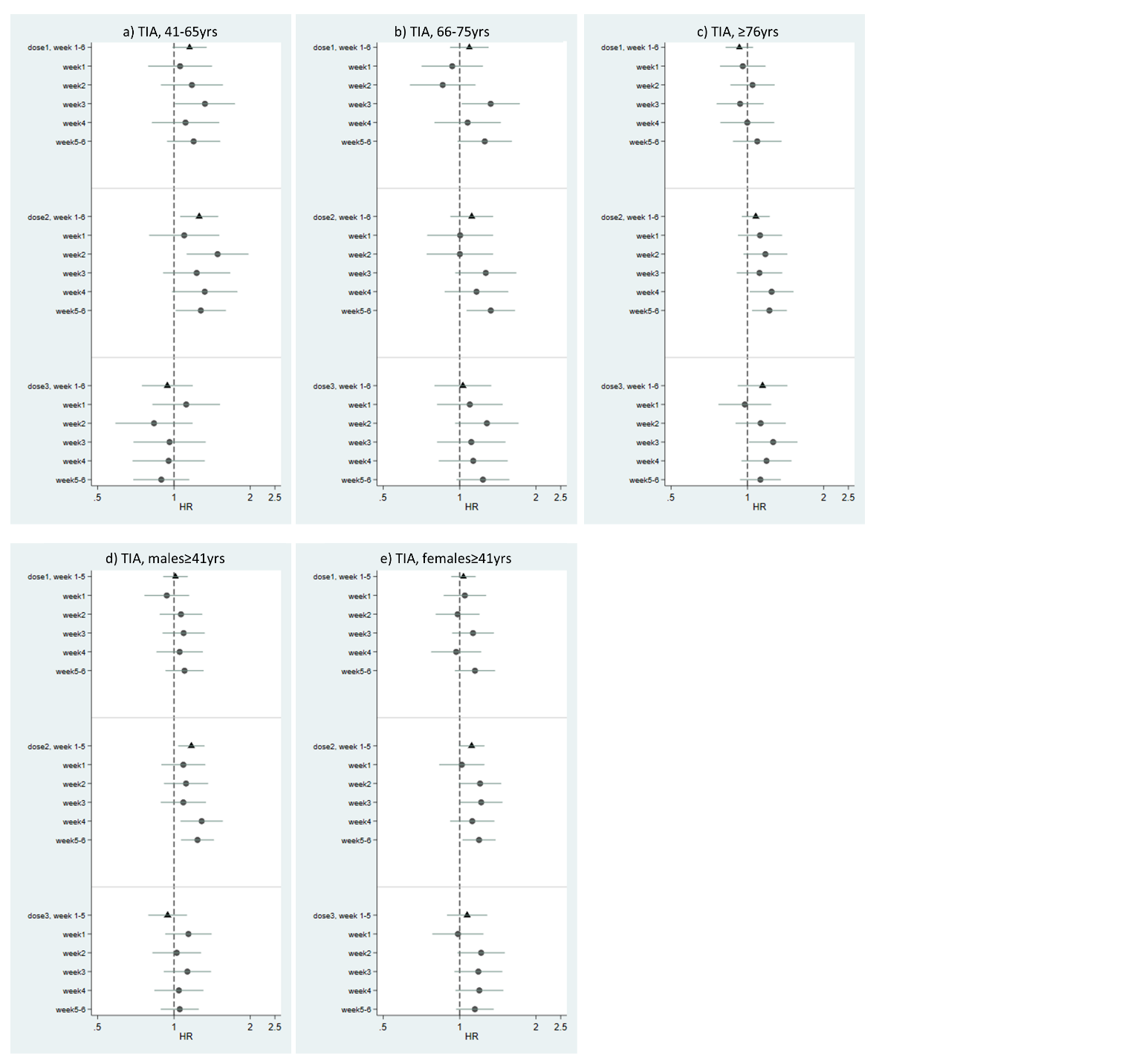
Figure S8. Forest plot showing hazard ratios (HR) for week 1-6 (triangle) and for each risk window (dot) with 95% confidence intervals (CI) for the **transit ischemic attack (TIA) in older age groups (a - c) and in males and females aged ≥ 41 years (d and e),** separately.

HR (triangle and dot) and 95%CI (lines) were obtained from a full adjusted model. For age groups 18-25yrs and 26-40yrs, there were too few cases (<5 cases) to provide HR estimates for each risk window.


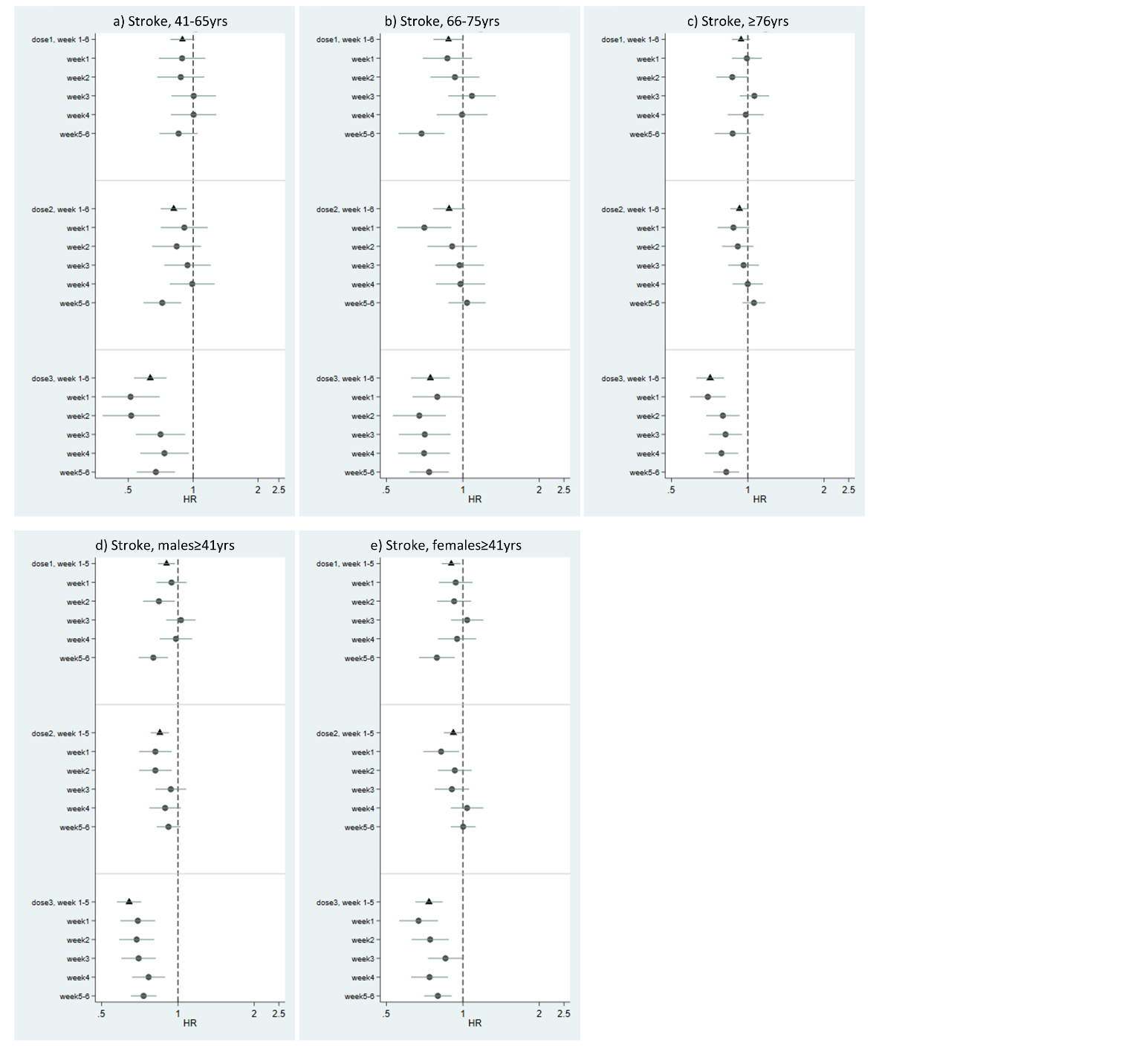


### Figure S9. Forest plot showing hazard ratios (HR) for week 1-6 (triangle) and for each risk window (dot) with 95% confidence intervals (CI) for the **stroke in older age groups (a - c) and in males and females aged ≥ 41 years (d and e),** separately.

HR (triangle and dot) and 95%CI (lines) were obtained from a full adjusted model. For age groups 18-25yrs and 26-40yrs, there were too few cases (<10 cases) to provide HR estimates for each risk window.


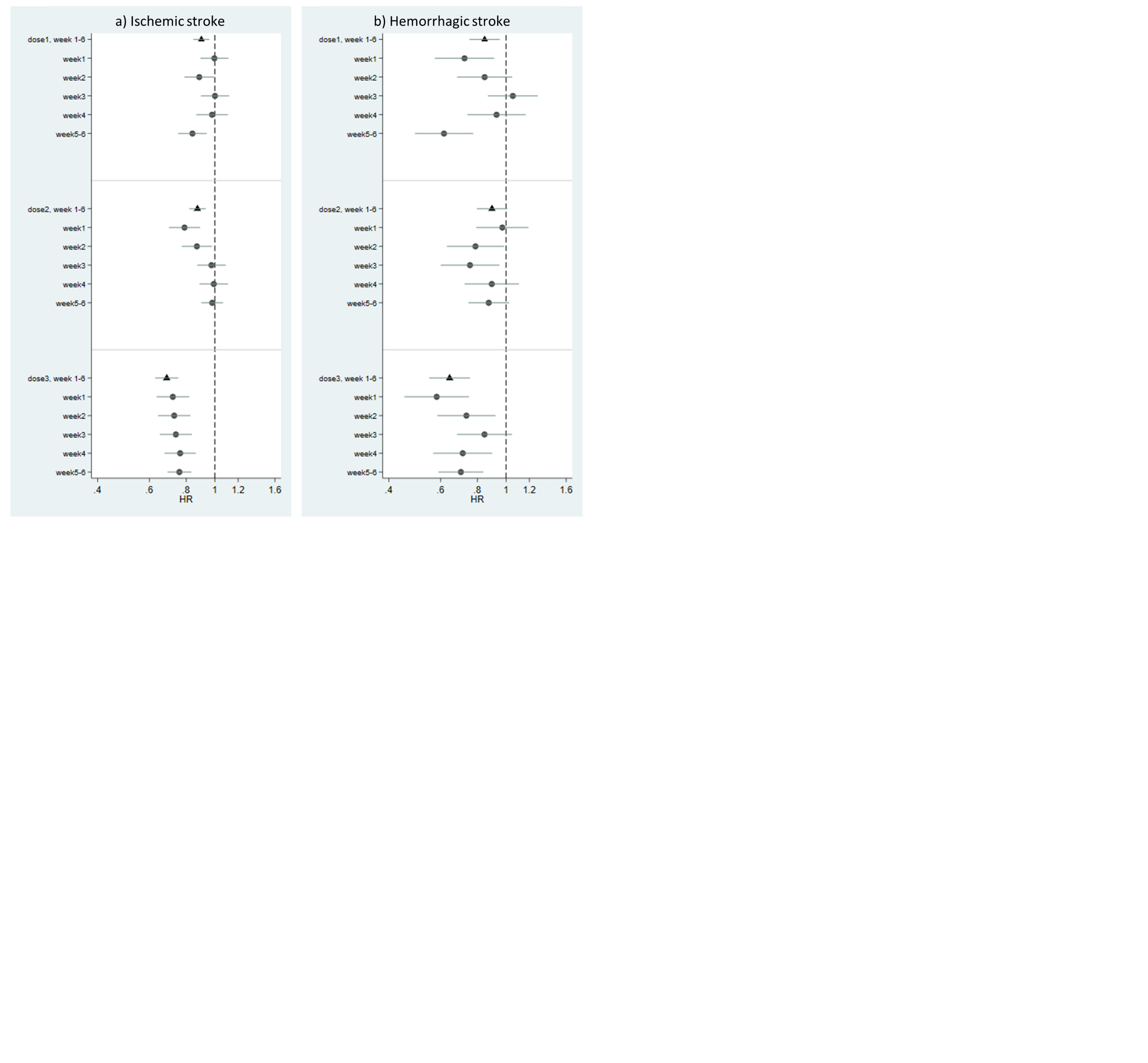


### Figure S10. Forest plot showing hazard ratios (HR) for week 1-6 (triangle) and for each risk window (dot) with 95% confidence intervals (CI) for the **ischemic stroke (a) and hemorrhagic stroke (b)**.

The detailed data are presented in Supplemental Table S6. HR (triangle and dot) and 95%CI (lines) were obtained from a full adjusted model.


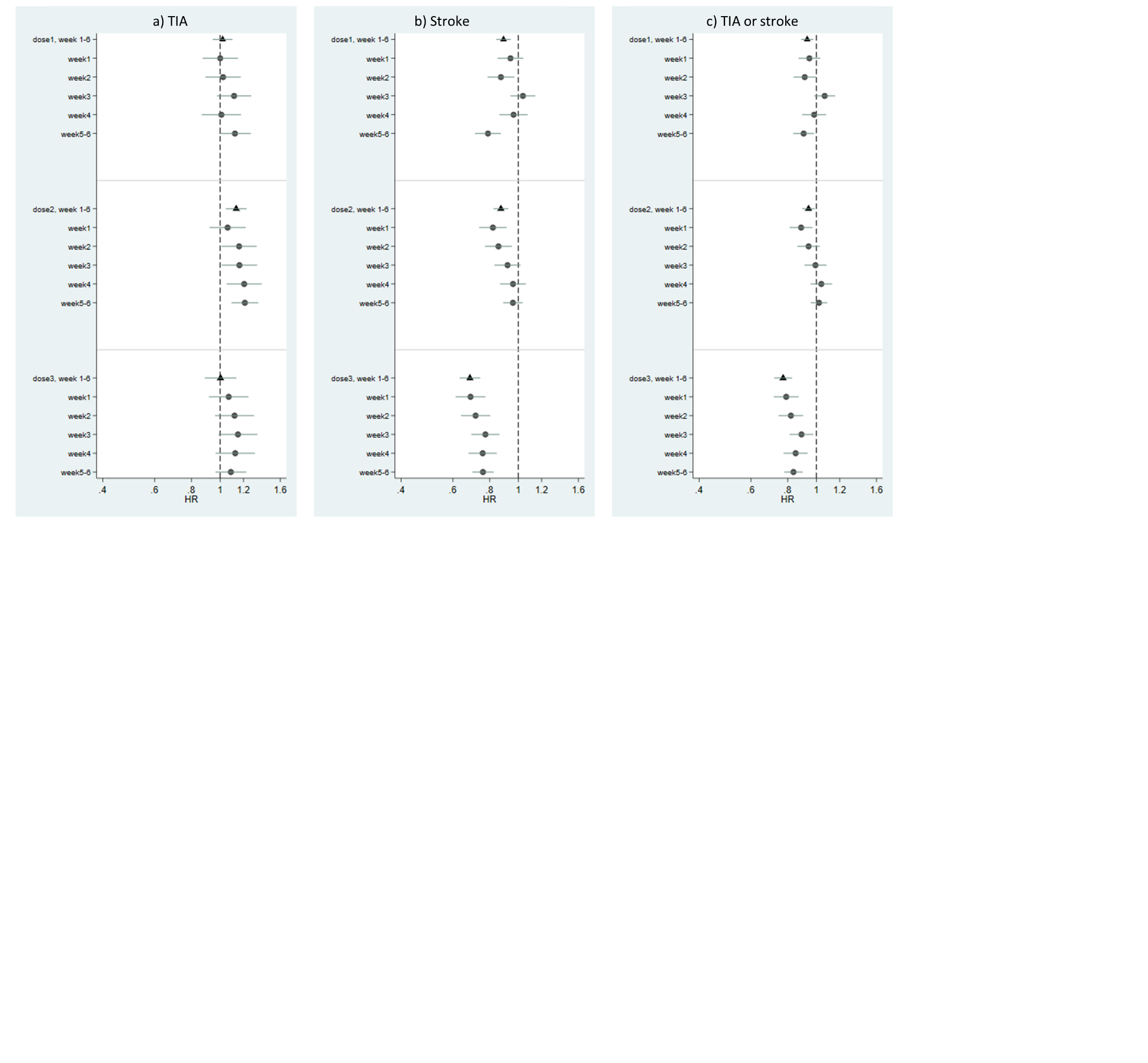


### Figure S11. Forest plot showing hazard ratios (HR) for week 1-6 (triangle) and for each risk window (dot) with 95% confidence intervals (CI) for the **transit ischemic attack (TIA, a) stroke (b) and the composite of TIA and stroke (c)**.

The detailed data are presented in Supplemental Table S5. HR (triangle and dot) and 95%CI (lines) were obtained from a full adjusted model.

## Supplemental Tables

### Table S1. List of prior comorbidities and treatments, and corresponding ICD-10-SE and ATC codes. The comorbidities were considered from 1 January 2015 to the date of study start, while the prior treatments were considered from 1 January 2018 to the date of study start

| Prior comorbidities and treatments | ICD-10 from NPR from 2015 | Prescription medication from NPDR from 2018 |
| --- | --- | --- |
| Hypertension (diagnosis or medication) | I10-I15 | C07 (except C07AA07), C02, C09A, C09B, C09C, C09D, C09X, C08C, C03A, C03EA01 |
| Diabetes (type 1 and 2) | E10, E11 | A10A, A10B |
| Chronic pulmonary disease | J430 J431 J432 J438 J439 J448 J449 J840 J841 J848 J849 J961 J969 E840 E841 E848 E849 |  |
| Asthma | J45 |  |
| Chronic kidney disease | N17-N19 |  |
| Cancer | C00-C97 |  |
| Autoimmune disease | M05-M14 |  |
| Thyroid disease | E00-E07 | H03 |
| Coagulation disorder | D65-D69 |  |
| Psychiatric disorders | F20 F21 F22 F23 F24 F25 F26 F27 F28 F29 F30 F31 F32 F33 F34 F35 F36 F37 F38 F39 |  |
| Antidepressant treatment |  | N06A |
| Obesity | E66 |  |

### Table S2. Hazard ratios (HR) with 95% confidence interval (CI) for **myocarditis and pericarditis** after each dose in each risk windows, among Swedish adults. Myocarditis and pericarditis used composite endpoints, including specialist outpatient visits, hospital admissions and deaths.

| Risk window | Person-years | Cases | Incidence rate  (Per 100 000 person-years) | Crude model, *  HR (95% CI) | Full model, †  HR (95% CI) |
| --- | --- | --- | --- | --- | --- |
| ***Myopericarditis*** |  |  |  |  |  |
| Unvaccinated | 4753813 | 1365 | 28.7 | Ref | Ref |
| Dose 1 |  |  |  |  |  |
| Week 1-6 | 759788 | 265 | 34.9 | 1.28 (1.11, 1.48) | 1.24 (1.06, 1.45) |
| Week1 | 136583 | 54 | 39.5 | 1.46 (1.11, 1.92) | 1.59 (1.2, 2.12) |
| Week2 | 136560 | 53 | 38.8 | 1.42 (1.08, 1.88) | 1.43 (1.06, 1.94) |
| Week3 | 136474 | 47 | 34.4 | 1.24 (0.92, 1.67) | 1.2 (0.87, 1.66) |
| Week4 | 128333 | 39 | 30.4 | 1.11 (0.80, 1.53) | 1.2 (0.86, 1.69) |
| Week5-6 | 221838 | 72 | 32.5 | 1.19 (0.93, 1.52) | 1.21 (0.92, 1.57) |
| Dose 2 |  |  |  |  |  |
| Week 1-6 | 804366 | 355 | 44.1 | 1.48 (1.29, 1.70) | 1.45 (1.25, 1.69) |
| Week1 | 134142 | 117 | 87.2 | 3.10 (2.55, 3.78) | 3.6 (2.94, 4.4) |
| Week2 | 134117 | 50 | 37.3 | 1.31 (0.98, 1.75) | 1.37 (1, 1.86) |
| Week3 | 134087 | 50 | 37.3 | 1.29 (0.97, 1.73) | 1.31 (0.96, 1.79) |
| Week4 | 134049 | 45 | 33.6 | 1.15 (0.85, 1.56) | 1.2 (0.87, 1.65) |
| Week5-6 | 267972 | 93 | 34.7 | 1.16 (0.93, 1.44) | 1.17 (0.92, 1.48) |
| Dose 3 |  |  |  |  |  |
| Week 1-6 | 628281 | 238 | 37.9 | 1.11 (0.92, 1.34) | 0.95 (0.78, 1.17) |
| Week1 | 104784 | 44 | 42.0 | 1.24 (0.90, 1.69) | 1.06 (0.76, 1.48) |
| Week2 | 104763 | 40 | 38.2 | 1.13 (0.81, 1.56) | 1.09 (0.79, 1.52) |
| Week3 | 104737 | 48 | 45.8 | 1.36 (1.00, 1.83) | 1.27 (0.93, 1.74) |
| Week4 | 104702 | 31 | 29.6 | 0.90 (0.62, 1.29) | 0.8 (0.55, 1.18) |
| Week5-6 | 209295 | 75 | 35.8 | 1.13 (0.88, 1.44) | 0.97 (0.74, 1.26) |
|  |  |  |  |  |  |
| ***Myocarditis*** |  |  |  |  |  |
| Unvaccinated | 4757083 | 628 | 13.2 | Ref | Ref |
| Dose1 |  |  |  |  |  |
| Week 1-6 | 760379 | 112 | 14.7 | 1.07 (0.86, 1.33) | 1.13 (0.9, 1.41) |
| Week1 | 136690 | 28 | 20.5 | 1.65 (1.12, 2.42) | 1.67 (1.14, 2.45) |
| Week2 | 136667 | 19 | 13.9 | 1.10 (0.69, 1.75) | 1.13 (0.71, 1.8) |
| Week3 | 136581 | 17 | 12.5 | 0.97 (0.60, 1.58) | 1.01 (0.62, 1.65) |
| Week4 | 128433 | 18 | 14.0 | 1.09 (0.68, 1.76) | 1.15 (0.72, 1.86) |
| Week5-6 | 222009 | 30 | 13.5 | 1.03 (0.71, 1.51) | 1.11 (0.76, 1.62) |
| Dose2 |  |  |  |  |  |
| Week 1-6 | 805012 | 177 | 22.0 | 1.32 (1.08, 1.60) | 1.43 (1.17, 1.75) |
| Week1 | 134248 | 79 | 58.9 | 4.33 (3.38, 5.55) | 4.62 (3.6, 5.93) |
| Week2 | 134224 | 23 | 17.1 | 1.22 (0.80, 1.87) | 1.3 (0.85, 2) |
| Week3 | 134194 | 17 | 12.7 | 0.87 (0.53, 1.41) | 0.93 (0.57, 1.51) |
| Week4 | 134157 | 21 | 15.6 | 1.04 (0.67, 1.62) | 1.12 (0.72, 1.74) |
| Week5-6 | 268189 | 37 | 13.8 | 0.88 (0.62, 1.24) | 0.95 (0.67, 1.34) |
| Dose3 |  |  |  |  |  |
| Week 1-6 | 628839 | 109 | 17.3 | 0.89 (0.69, 1.17) | 0.91 (0.68, 1.2) |
| Week1 | 104876 | 18 | 17.2 | 0.82 (0.50, 1.32) | 0.89 (0.55, 1.45) |
| Week2 | 104856 | 24 | 22.9 | 1.09 (0.71, 1.66) | 1.22 (0.8, 1.88) |
| Week3 | 104830 | 16 | 15.3 | 0.73 (0.44, 1.22) | 0.84 (0.5, 1.4) |
| Week4 | 104795 | 17 | 16.2 | 0.79 (0.48, 1.30) | 0.91 (0.55, 1.49) |
| Week5-6 | 209482 | 34 | 16.2 | 0.80 (0.56, 1.15) | 0.91 (0.63, 1.32) |
|  |  |  |  |  |  |
| ***Pericarditis*** |  |  |  |  |  |
| Unvaccinated | 4757536 | 642 | 13.5 | Ref | Ref |
| Dose1 |  |  |  |  |  |
| Week 1-6 | 760358 | 138 | 18.2 | 1.47 (1.20, 1.81) | 1.31 (1.06, 1.61) |
| Week1 | 136685 | 29 | 21.2 | 1.73 (1.18, 2.52) | 1.64 (1.12, 2.39) |
| Week2 | 136662 | 30 | 22.0 | 1.78 (1.23, 2.58) | 1.69 (1.16, 2.46) |
| Week3 | 136576 | 23 | 16.8 | 1.33 (0.87, 2.03) | 1.26 (0.83, 1.93) |
| Week4 | 128431 | 20 | 15.6 | 1.23 (0.78, 1.93) | 1.19 (0.76, 1.88) |
| Week5-6 | 222005 | 36 | 16.2 | 1.28 (0.90, 1.81) | 1.28 (0.9, 1.81) |
| Dose2 |  |  |  |  |  |
| Week 1-6 | 804977 | 178 | 22.1 | 1.73 (1.42, 2.11) | 1.48 (1.21, 1.82) |
| Week1 | 134243 | 48 | 35.8 | 2.77 (2.04, 3.75) | 2.7 (1.99, 3.66) |
| Week2 | 134218 | 27 | 20.1 | 1.58 (1.07, 2.35) | 1.54 (1.04, 2.29) |
| Week3 | 134188 | 28 | 20.9 | 1.66 (1.12, 2.45) | 1.61 (1.09, 2.37) |
| Week4 | 134151 | 24 | 17.9 | 1.42 (0.93, 2.15) | 1.37 (0.9, 2.08) |
| Week5-6 | 268177 | 51 | 19.0 | 1.47 (1.09, 1.99) | 1.41 (1.04, 1.91) |
| Dose3 |  |  |  |  |  |
| Week 1-6 | 628754 | 128 | 20.4 | 1.43 (1.09, 1.88) | 0.98 (0.74, 1.3) |
| Week1 | 104862 | 22 | 21.0 | 1.45 (0.93, 2.26) | 1.1 (0.71, 1.72) |
| Week2 | 104842 | 19 | 18.1 | 1.28 (0.80, 2.06) | 0.99 (0.61, 1.59) |
| Week3 | 104816 | 33 | 31.5 | 2.20 (1.52, 3.19) | 1.71 (1.17, 2.49) |
| Week4 | 104781 | 15 | 14.3 | 1.00 (0.59, 1.70) | 0.77 (0.45, 1.31) |
| Week5-6 | 209454 | 39 | 18.6 | 1.38 (0.97, 1.95) | 1.04 (0.73, 1.48) |

*Crude model included no covariates.

†Full model included age, sex, country of birth, employed as a healthcare worker, marital status, education, COVID-19 infection, and health seeking behaviours during 2018-19 (ie, no. of primary care visits, number of specialist outpatient visits), and prior comorbidities and treatments listed in Supplemental Table S1.

### Table S3. Hazard ratios (HR) with 95% confidence interval (CI) for **dysrhythmia** after each dose in each risk windows, among Swedish adults. All dysrhythmia used composite endpoints, including specialist outpatient visits, hospital admissions and deaths.

| Risk window | Person-years | Cases | Incidence rate  (Per 100 000 person-years) | Crude model, *  HR (95% CI) | Full model, †  HR (95% CI) |
| --- | --- | --- | --- | --- | --- |
| ***Extrasystoles*** |  |  |  |  |  |
| Unvaccinated | 4746214 | 2858 | 60.2 | Ref | Ref |
| Dose1 |  |  |  |  |  |
| Week 1-6 | 757458 | 701 | 92.6 | 1.91 (1.74, 2.09) | 1.17 (1.06, 1.28) |
| Week1 | 136159 | 130 | 95.5 | 1.61 (1.35, 1.92) | 1.16 (0.97, 1.39) |
| Week2 | 136134 | 139 | 102.1 | 1.78 (1.50, 2.11) | 1.26 (1.06, 1.5) |
| Week3 | 136047 | 111 | 81.6 | 1.48 (1.22, 1.79) | 1.03 (0.85, 1.25) |
| Week4 | 127945 | 104 | 81.3 | 1.55 (1.27, 1.90) | 1.09 (0.89, 1.33) |
| Week5-6 | 221173 | 217 | 98.1 | 1.96 (1.69, 2.27) | 1.36 (1.17, 1.58) |
| Dose2 |  |  |  |  |  |
| Week 1-6 | 801744 | 698 | 87.1 | 2.33 (2.11, 2.57) | 1.22 (1.1, 1.36) |
| Week1 | 133708 | 135 | 101.0 | 1.98 (1.66, 2.36) | 1.33 (1.11, 1.59) |
| Week2 | 133682 | 116 | 86.8 | 1.74 (1.44, 2.11) | 1.17 (0.96, 1.42) |
| Week3 | 133650 | 114 | 85.3 | 1.78 (1.47, 2.15) | 1.19 (0.98, 1.44) |
| Week4 | 133612 | 113 | 84.6 | 1.83 (1.51, 2.22) | 1.22 (1, 1.48) |
| Week5-6 | 267093 | 220 | 82.4 | 1.89 (1.64, 2.19) | 1.25 (1.08, 1.45) |
| Dose3 |  |  |  |  |  |
| Week 1-6 | 625513 | 617 | 98.6 | 2.51 (2.18, 2.89) | 0.98 (0.84, 1.14) |
| Week1 | 104326 | 117 | 112.2 | 3.35 (2.75, 4.09) | 1.22 (1, 1.49) |
| Week2 | 104303 | 98 | 94.0 | 2.99 (2.41, 3.71) | 1.07 (0.87, 1.33) |
| Week3 | 104276 | 116 | 111.2 | 3.70 (3.03, 4.53) | 1.31 (1.07, 1.61) |
| Week4 | 104240 | 86 | 82.5 | 2.75 (2.19, 3.45) | 0.95 (0.76, 1.2) |
| Week5-6 | 208368 | 200 | 96.0 | 3.27 (2.78, 3.84) | 1.08 (0.92, 1.27) |
|  |  |  |  |  |  |
| ***Atrial fibrillation*** |  |  |  |  |  |
| Unvaccinated | 4670230 | 15825 | 338.9 | Ref | Ref |
| Dose1 |  |  |  |  |  |
| Week 1-6 | 734493 | 4122 | 561.2 | 2.73 (2.62, 2.83) | 0.9 (0.87, 0.94) |
| Week1 | 131502 | 743 | 565.0 | 1.86 (1.72, 2.00) | 0.87 (0.8, 0.94) |
| Week2 | 131472 | 841 | 639.7 | 2.21 (2.06, 2.37) | 0.97 (0.91, 1.05) |
| Week3 | 131385 | 827 | 629.5 | 2.31 (2.15, 2.48) | 0.97 (0.9, 1.04) |
| Week4 | 124281 | 677 | 544.7 | 2.19 (2.02, 2.37) | 0.97 (0.89, 1.05) |
| Week5-6 | 215853 | 1034 | 479.0 | 2.13 (2.00, 2.28) | 0.99 (0.92, 1.06) |
| Dose2 |  |  |  |  |  |
| Week 1-6 | 773694 | 4776 | 617.3 | 4.67 (4.49, 4.86) | 0.92 (0.88, 0.96) |
| Week1 | 129046 | 781 | 605.2 | 2.77 (2.57, 2.98) | 0.94 (0.87, 1.01) |
| Week2 | 129014 | 805 | 624.0 | 3.00 (2.79, 3.23) | 0.98 (0.91, 1.06) |
| Week3 | 128977 | 854 | 662.1 | 3.35 (3.12, 3.60) | 1.05 (0.97, 1.12) |
| Week4 | 128933 | 776 | 601.9 | 3.22 (2.99, 3.47) | 0.96 (0.89, 1.03) |
| Week5-6 | 257724 | 1560 | 605.3 | 3.52 (3.33, 3.73) | 0.99 (0.94, 1.05) |
| Dose3 |  |  |  |  |  |
| Week 1-6 | 597926 | 4560 | 762.6 | 5.56 (5.25, 5.89) | 0.8 (0.75, 0.85) |
| Week1 | 99740 | 704 | 705.8 | 10.00 (9.20, 10.87) | 0.78 (0.72, 0.85) |
| Week2 | 99712 | 760 | 762.2 | 11.44 (10.55, 12.41) | 0.84 (0.78, 0.91) |
| Week3 | 99680 | 763 | 765.5 | 12.26 (11.31, 13.30) | 0.84 (0.77, 0.91) |
| Week4 | 99639 | 780 | 782.8 | 13.37 (12.33, 14.49) | 0.86 (0.79, 0.93) |
| Week5-6 | 199156 | 1553 | 779.8 | 14.82 (13.89, 15.80) | 0.86 (0.81, 0.92) |
|  |  |  |  |  |  |
| ***Arrhythmias*** |  |  |  |  |  |
| Unvaccinated | 4607898 | 27193 | 590.1 | Ref | Ref |
| Dose 1 |  |  |  |  |  |
| Week 1-6 | 721219 | 6334 | 878.2 | 2.24 (2.17, 2.31) | 0.95 (0.92, 0.98) |
| Week1 | 129043 | 1138 | 881.9 | 1.54 (1.45, 1.64) | 0.89 (0.84, 0.95) |
| Week2 | 129007 | 1282 | 993.8 | 1.81 (1.71, 1.91) | 1 (0.95, 1.06) |
| Week3 | 128916 | 1229 | 953.3 | 1.81 (1.71, 1.92) | 0.97 (0.92, 1.03) |
| Week4 | 122072 | 1043 | 854.4 | 1.73 (1.63, 1.85) | 0.99 (0.93, 1.05) |
| Week5-6 | 212180 | 1642 | 773.9 | 1.69 (1.6, 1.78) | 1 (0.95, 1.06) |
| Dose 2 |  |  |  |  |  |
| Week 1-6 | 758790 | 7099 | 935.6 | 3.37 (3.27, 3.48) | 0.98 (0.95, 1.02) |
| Week1 | 126577 | 1227 | 969.4 | 2.17 (2.05, 2.30) | 1.01 (0.96, 1.08) |
| Week2 | 126538 | 1223 | 966.5 | 2.24 (2.11, 2.38) | 1.02 (0.96, 1.08) |
| Week3 | 126495 | 1223 | 966.8 | 2.33 (2.20, 2.47) | 1.03 (0.97, 1.09) |
| Week4 | 126445 | 1163 | 919.8 | 2.31 (2.17, 2.45) | 0.99 (0.93, 1.05) |
| Week5-6 | 252735 | 2263 | 895.4 | 2.38 (2.28, 2.50) | 0.98 (0.94, 1.03) |
| Dose 3 |  |  |  |  |  |
| Week 1-6 | 584095 | 6591 | 1128.4 | 3.53 (3.38, 3.69) | 0.81 (0.77, 0.85) |
| Week1 | 97446 | 1075 | 1103.2 | 5.18 (4.85, 5.54) | 0.86 (0.81, 0.92) |
| Week2 | 97413 | 1105 | 1134.4 | 5.56 (5.21, 5.94) | 0.89 (0.83, 0.94) |
| Week3 | 97376 | 1112 | 1142.0 | 5.85 (5.47, 6.24) | 0.89 (0.84, 0.95) |
| Week4 | 97332 | 1070 | 1099.3 | 5.84 (5.46, 6.24) | 0.86 (0.8, 0.92) |
| Week5-6 | 194528 | 2229 | 1145.9 | 6.52 (6.19, 6.86) | 0.89 (0.85, 0.94) |

*Crude model included no covariates.

†Full model included age, sex, country of birth, employed as a healthcare worker, marital status, education, COVID-19 infection, and health seeking behaviours during 2018-19 (i.e., no. of primary care visits, number of specialist outpatient visits), and prior comorbidities and treatments listed in Supplemental Table S1.

### Table S4. Hazard ratios (HR) with 95% confidence interval (CI) for **cardiac outcomes** after each dose in each risk windows, among Swedish adults. All cardiac outcomes used composite endpoints, including specialist outpatient visits, hospital admissions and deaths.

| Risk window | Person-years | Cases | Incidence rate  (Per 100 000 person-years) | Crude model, *  HR (95% CI) | Full model, †  HR (95% CI) |
| --- | --- | --- | --- | --- | --- |
| ***Myocardial infarction*** |  |  |  |  |  |
| Unvaccinated | 4727981 | 8109 | 171.5 | Ref | Ref |
| Dose1 |  |  |  |  |  |
| Week 1-6 | 752452 | 1918 | 254.9 | 2.25 (2.13, 2.39) | 0.95 (0.89, 1.01) |
| Week1 | 135171 | 352 | 260.4 | 1.59 (1.43, 1.77) | 0.94 (0.84, 1.06) |
| Week2 | 135144 | 380 | 281.2 | 1.78 (1.60, 1.97) | 0.99 (0.88, 1.11) |
| Week3 | 135056 | 381 | 282.1 | 1.85 (1.67, 2.05) | 0.99 (0.88, 1.11) |
| Week4 | 127146 | 329 | 258.8 | 1.81 (1.62, 2.03) | 1.03 (0.91, 1.16) |
| Week5-6 | 219934 | 476 | 216.4 | 1.64 (1.49, 1.81) | 0.99 (0.89, 1.09) |
| Dose2 |  |  |  |  |  |
| Week 1-6 | 795761 | 2065 | 259.5 | 3.13 (2.95, 3.33) | 0.91 (0.85, 0.98) |
| Week1 | 132715 | 315 | 237.4 | 1.84 (1.64, 2.07) | 0.89 (0.79, 1.01) |
| Week2 | 132687 | 339 | 255.5 | 2.06 (1.84, 2.30) | 0.94 (0.83, 1.06) |
| Week3 | 132654 | 346 | 260.8 | 2.17 (1.94, 2.42) | 0.94 (0.84, 1.06) |
| Week4 | 132614 | 376 | 283.5 | 2.44 (2.20, 2.72) | 1.09 (0.98, 1.22) |
| Week5-6 | 265092 | 689 | 259.9 | 2.35 (2.17, 2.56) | 0.95 (0.87, 1.04) |
| Dose3 |  |  |  |  |  |
| Week 1-6 | 619549 | 1974 | 318.6 | 3.44 (3.16, 3.74) | 0.81 (0.74, 0.89) |
| Week1 | 103337 | 319 | 308.7 | 5.55 (4.91, 6.27) | 0.83 (0.73, 0.94) |
| Week2 | 103312 | 308 | 298.1 | 5.48 (4.84, 6.20) | 0.8 (0.7, 0.91) |
| Week3 | 103283 | 320 | 309.8 | 5.95 (5.27, 6.72) | 0.83 (0.73, 0.94) |
| Week4 | 103245 | 369 | 357.4 | 7.29 (6.49, 8.18) | 0.95 (0.85, 1.07) |
| Week5-6 | 206373 | 658 | 318.8 | 7.10 (6.46, 7.80) | 0.84 (0.77, 0.93) |
|  |  |  |  |  |  |
| ***Heart failure*** |  |  |  |  |  |
| Unvaccinated | 4713312 | 12411 | 263.3 | Ref | Ref |
| Dose1 |  |  |  |  |  |
| Week 1-6 | 747862 | 3008 | 402.2 | 2.74 (2.62, 2.86) | 0.86 (0.82, 0.9) |
| Week1 | 134079 | 547 | 408.0 | 1.72 (1.58, 1.88) | 0.78 (0.71, 0.85) |
| Week2 | 134051 | 630 | 470.0 | 2.09 (1.92, 2.27) | 0.89 (0.82, 0.97) |
| Week3 | 133965 | 654 | 488.2 | 2.30 (2.12, 2.49) | 0.93 (0.86, 1.01) |
| Week4 | 126475 | 517 | 408.8 | 2.11 (1.92, 2.31) | 0.94 (0.86, 1.03) |
| Week5-6 | 219293 | 660 | 301.0 | 1.73 (1.59, 1.88) | 0.86 (0.8, 0.94) |
| Dose2 |  |  |  |  |  |
| Week 1-6 | 789255 | 3689 | 467.4 | 4.69 (4.49, 4.90) | 0.88 (0.84, 0.93) |
| Week1 | 131634 | 603 | 458.1 | 2.69 (2.47, 2.93) | 0.88 (0.81, 0.96) |
| Week2 | 131604 | 595 | 452.1 | 2.79 (2.56, 3.03) | 0.87 (0.8, 0.95) |
| Week3 | 131570 | 609 | 462.9 | 3.01 (2.77, 3.28) | 0.9 (0.83, 0.98) |
| Week4 | 131528 | 634 | 482.0 | 3.32 (3.06, 3.61) | 0.95 (0.88, 1.03) |
| Week5-6 | 262919 | 1248 | 474.7 | 3.55 (3.33, 3.78) | 0.95 (0.89, 1.01) |
| Dose3 |  |  |  |  |  |
| Week 1-6 | 613255 | 3790 | 618.0 | 4.93 (4.64, 5.24) | 0.73 (0.69, 0.78) |
| Week1 | 102291 | 560 | 547.5 | 9.25 (8.42, 10.15) | 0.72 (0.66, 0.79) |
| Week2 | 102265 | 629 | 615.1 | 10.84 (9.91, 11.85) | 0.79 (0.73, 0.87) |
| Week3 | 102234 | 649 | 634.8 | 11.80 (10.80, 12.90) | 0.81 (0.75, 0.88) |
| Week4 | 102195 | 643 | 629.2 | 12.31 (11.26, 13.46) | 0.79 (0.73, 0.86) |
| Week5-6 | 204271 | 1309 | 640.8 | 13.79 (12.85, 14.79) | 0.8 (0.75, 0.85) |

*Crude model included no covariates.

†Full model included age, sex, country of birth, employed as a healthcare worker, marital status, education, COVID-19 infection, and health seeking behaviours during 2018-19 (i.e., no. of primary care visits, number of specialist outpatient visits), and prior comorbidities and treatments listed in Supplemental Table S1.

### Table S5. Hazard ratios (HR) with 95% confidence interval (CI) for **transit ischemic attack (TIA) and stroke** after each dose in each risk windows, among Swedish adults. TIA and stroke used composite endpoints, including specialist outpatient visits, hospital admissions and deaths.

| Risk window | Person-years | Cases | Incidence rate  (Per 100 000 person-years) | Crude model, *  HR (95% CI) | Full model, †  HR (95% CI) |
| --- | --- | --- | --- | --- | --- |
| ***TIA*** |  |  |  |  |  |
| Unvaccinated | 4737698 | 4463 | 94.2 | Ref | Ref |
| Dose1 |  |  |  |  |  |
| Week 1-6 | 754308 | 1212 | 160.7 | 2.59 (2.41, 2.78) | 1.02 (0.94, 1.1) |
| Week1 | 135502 | 225 | 166.1 | 1.92 (1.68, 2.20) | 1 (0.87, 1.15) |
| Week2 | 135476 | 228 | 168.3 | 2.04 (1.78, 2.33) | 1.02 (0.89, 1.17) |
| Week3 | 135389 | 249 | 183.9 | 2.33 (2.04, 2.65) | 1.12 (0.98, 1.27) |
| Week4 | 127449 | 186 | 145.9 | 2.02 (1.74, 2.35) | 1.01 (0.87, 1.18) |
| Week5-6 | 220491 | 324 | 146.9 | 2.25 (2.00, 2.53) | 1.12 (0.99, 1.27) |
| Dose2 |  |  |  |  |  |
| Week 1-6 | 797756 | 1398 | 175.2 | 4.38 (4.08, 4.7) | 1.13 (1.05, 1.23) |
| Week1 | 133046 | 216 | 162.4 | 2.54 (2.2, 2.92) | 1.06 (0.92, 1.22) |
| Week2 | 133019 | 236 | 177.4 | 2.90 (2.54, 3.33) | 1.16 (1.01, 1.33) |
| Week3 | 132986 | 233 | 175.2 | 3.01 (2.63, 3.45) | 1.16 (1.01, 1.34) |
| Week4 | 132946 | 244 | 183.5 | 3.30 (2.88, 3.77) | 1.21 (1.05, 1.38) |
| Week5-6 | 265758 | 469 | 176.5 | 3.40 (3.06, 3.77) | 1.21 (1.09, 1.35) |
| Dose3 |  |  |  |  |  |
| Week 1-6 | 621352 | 1229 | 197.8 | 5.40 (4.82, 6.04) | 1 (0.89, 1.14) |
| Week1 | 103635 | 201 | 194.0 | 9.18 (7.86, 10.73) | 1.07 (0.92, 1.25) |
| Week2 | 103611 | 208 | 200.8 | 10.04 (8.61, 11.71) | 1.12 (0.96, 1.3) |
| Week3 | 103583 | 212 | 204.7 | 10.82 (9.28, 12.61) | 1.15 (0.99, 1.34) |
| Week4 | 103546 | 205 | 198.0 | 11.08 (9.48, 12.95) | 1.13 (0.97, 1.31) |
| Week5-6 | 206978 | 403 | 194.7 | 11.73 (10.37, 13.27) | 1.09 (0.96, 1.23) |
|  |  |  |  |  |  |
| ***Stroke*** |  |  |  |  |  |
| Unvaccinated | 4725902 | 9236 | 195.4 | Ref | Ref |
| Dose1 |  |  |  |  |  |
| Week 1-6 | 751741 | 2163 | 287.7 | 2.31 (2.18, 2.43) | 0.89 (0.84, 0.94) |
| Week1 | 134913 | 430 | 318.7 | 1.74 (1.58, 1.92) | 0.94 (0.85, 1.04) |
| Week2 | 134886 | 407 | 301.7 | 1.71 (1.55, 1.89) | 0.87 (0.79, 0.97) |
| Week3 | 134800 | 493 | 365.7 | 2.16 (1.97, 2.37) | 1.04 (0.94, 1.14) |
| Week4 | 127070 | 370 | 291.2 | 1.84 (1.66, 2.05) | 0.96 (0.86, 1.08) |
| Week5-6 | 220072 | 463 | 210.4 | 1.43 (1.30, 1.58) | 0.79 (0.71, 0.87) |
| Dose2 |  |  |  |  |  |
| Week 1-6 | 794247 | 2567 | 323.2 | 3.56 (3.38, 3.76) | 0.87 (0.82, 0.92) |
| Week1 | 132464 | 384 | 289.9 | 2.02 (1.82, 2.24) | 0.82 (0.74, 0.91) |
| Week2 | 132435 | 398 | 300.5 | 2.18 (1.97, 2.42) | 0.86 (0.77, 0.95) |
| Week3 | 132402 | 442 | 333.8 | 2.54 (2.30, 2.80) | 0.92 (0.83, 1.02) |
| Week4 | 132361 | 451 | 340.7 | 2.72 (2.46, 3.00) | 0.96 (0.87, 1.06) |
| Week5-6 | 264585 | 892 | 337.1 | 2.87 (2.67, 3.09) | 0.96 (0.89, 1.03) |
| Dose3 |  |  |  |  |  |
| Week 1-6 | 618096 | 2325 | 376.2 | 3.54 (3.29, 3.82) | 0.69 (0.63, 0.74) |
| Week1 | 103094 | 348 | 337.6 | 6.17 (5.50, 6.94) | 0.69 (0.61, 0.77) |
| Week2 | 103070 | 371 | 360.0 | 6.88 (6.14, 7.71) | 0.72 (0.64, 0.8) |
| Week3 | 103040 | 396 | 384.3 | 7.68 (6.88, 8.58) | 0.77 (0.69, 0.86) |
| Week4 | 103003 | 397 | 385.4 | 8.07 (7.22, 9.02) | 0.76 (0.68, 0.85) |
| Week5-6 | 205890 | 813 | 394.9 | 8.91 (8.17, 9.71) | 0.76 (0.7, 0.83) |
|  |  |  |  |  |  |
| ***TIA or stroke*** |  |  |  |  |  |
| Unvaccinated | 4706205 | 12144 | 258.0 | Ref | Ref |
| Dose1 |  |  |  |  |  |
| Week 1-6 | 746162 | 2969 | 397.9 | 2.40 (2.29, 2.51) | 0.93 (0.89, 0.98) |
| Week1 | 133844 | 572 | 427.4 | 1.74 (1.60, 1.90) | 0.95 (0.87, 1.03) |
| Week2 | 133815 | 552 | 412.5 | 1.74 (1.60, 1.90) | 0.91 (0.84, 1) |
| Week3 | 133727 | 643 | 480.8 | 2.11 (1.95, 2.29) | 1.07 (0.98, 1.16) |
| Week4 | 126157 | 491 | 389.2 | 1.83 (1.67, 2.01) | 0.98 (0.9, 1.08) |
| Week5-6 | 218619 | 711 | 325.2 | 1.64 (1.52, 1.78) | 0.91 (0.83, 0.98) |
| Dose2 |  |  |  |  |  |
| Week 1-6 | 787725 | 3450 | 438.0 | 3.78 (3.61, 3.96) | 0.94 (0.9, 0.99) |
| Week1 | 131383 | 527 | 401.1 | 2.08 (1.91, 2.28) | 0.89 (0.81, 0.97) |
| Week2 | 131352 | 556 | 423.3 | 2.29 (2.10, 2.50) | 0.94 (0.86, 1.03) |
| Week3 | 131316 | 583 | 444.0 | 2.51 (2.30, 2.73) | 0.99 (0.91, 1.08) |
| Week4 | 131273 | 605 | 460.9 | 2.72 (2.50, 2.96) | 1.04 (0.95, 1.13) |
| Week5-6 | 262401 | 1179 | 449.3 | 2.82 (2.64, 3.01) | 1.02 (0.96, 1.09) |
| Dose3 |  |  |  |  |  |
| Week 1-6 | 611535 | 3092 | 505.6 | 3.98 (3.73, 4.25) | 0.77 (0.72, 0.83) |
| Week1 | 102006 | 480 | 470.6 | 6.20 (5.62, 6.85) | 0.79 (0.72, 0.87) |
| Week2 | 101979 | 498 | 488.3 | 6.75 (6.12, 7.44) | 0.82 (0.74, 0.9) |
| Week3 | 101948 | 544 | 533.6 | 7.70 (7.01, 8.47) | 0.89 (0.81, 0.98) |
| Week4 | 101908 | 523 | 513.2 | 7.73 (7.02, 8.52) | 0.85 (0.77, 0.93) |
| Week5-6 | 203695 | 1047 | 514.0 | 8.26 (7.66, 8.90) | 0.84 (0.78, 0.9) |

*Crude model included no covariates.

†Full model included age, sex, country of birth, employed as a healthcare worker, marital status, education, COVID-19 infection, and health seeking behaviours during 2018-19 (i.e., no. of primary care visits, number of specialist outpatient visits), and prior comorbidities and treatments listed in Supplemental Table S1.

### Table S6. Hazard ratios (HR) with 95% confidence interval (CI) for **ischemic stroke and hemorrhagic stroke** after each dose in each risk windows, among Swedish adults. Both strokes used composite endpoints, including specialist outpatient visits, hospital admissions and deaths.

| Risk window | Person-years | Cases | Incidence rate  (Per 100 000 person-years) | Crude model, *  HR (95% CI) | Full model, †  HR (95% CI) |
| --- | --- | --- | --- | --- | --- |
| ***Ischemic stroke*** |  |  |  |  |  |
| Unvaccinated | 4732923 | 7012 | 148.2 | Ref | Ref |
| Dose1 |  |  |  |  |  |
| Week 1-6 | 753460 | 1664 | 220.9 | 2.36 (2.23, 2.51) | 0.9 (0.85, 0.96) |
| Week1 | 135264 | 348 | 257.3 | 1.83 (1.64, 2.04) | 1 (0.89, 1.11) |
| Week2 | 135238 | 310 | 229.2 | 1.68 (1.50, 1.89) | 0.89 (0.79, 0.99) |
| Week3 | 135151 | 350 | 259.0 | 1.97 (1.76, 2.20) | 1 (0.9, 1.12) |
| Week4 | 127341 | 280 | 219.9 | 1.78 (1.58, 2.01) | 0.98 (0.87, 1.11) |
| Week5-6 | 220465 | 376 | 170.6 | 1.47 (1.32, 1.64) | 0.84 (0.75, 0.94) |
| Dose2 |  |  |  |  |  |
| Week 1-6 | 796362 | 1971 | 247.5 | 3.68 (3.47, 3.91) | 0.87 (0.82, 0.93) |
| Week1 | 132815 | 282 | 212.3 | 1.87 (1.66, 2.12) | 0.79 (0.7, 0.89) |
| Week2 | 132787 | 309 | 232.7 | 2.14 (1.91, 2.41) | 0.87 (0.77, 0.98) |
| Week3 | 132754 | 346 | 260.6 | 2.51 (2.24, 2.80) | 0.97 (0.87, 1.09) |
| Week4 | 132714 | 348 | 262.2 | 2.65 (2.37, 2.96) | 0.99 (0.89, 1.11) |
| Week5-6 | 265292 | 686 | 258.6 | 2.78 (2.55, 3.02) | 0.98 (0.9, 1.07) |
| Dose3 |  |  |  |  |  |
| Week 1-6 | 620119 | 1787 | 288.2 | 3.66 (3.37, 3.99) | 0.69 (0.63, 0.75) |
| Week1 | 103430 | 279 | 269.8 | 5.84 (5.13, 6.65) | 0.72 (0.63, 0.82) |
| Week2 | 103406 | 284 | 274.7 | 6.19 (5.44, 7.05) | 0.73 (0.64, 0.83) |
| Week3 | 103377 | 293 | 283.4 | 6.60 (5.80, 7.50) | 0.74 (0.65, 0.84) |
| Week4 | 103340 | 308 | 298.1 | 7.21 (6.36, 8.17) | 0.76 (0.67, 0.86) |
| Week5-6 | 206566 | 623 | 301.6 | 7.82 (7.09, 8.63) | 0.76 (0.69, 0.83) |
|  |  |  |  |  |  |
| ***Hemorrhagic stroke*** |  |  |  |  |  |
| Unvaccinated | 4754027 | 2131 | 44.8 | Ref | Ref |
| Dose1 |  |  |  |  |  |
| Week 1-6 | 759225 | 445 | 58.6 | 2.05 (1.83, 2.29) | 0.85 (0.75, 0.95) |
| Week1 | 136433 | 76 | 55.7 | 1.26 (1.00, 1.59) | 0.72 (0.57, 0.91) |
| Week2 | 136410 | 90 | 66.0 | 1.52 (1.23, 1.88) | 0.85 (0.68, 1.05) |
| Week3 | 136324 | 112 | 82.2 | 1.97 (1.62, 2.39) | 1.05 (0.87, 1.28) |
| Week4 | 128262 | 82 | 63.9 | 1.62 (1.29, 2.03) | 0.93 (0.74, 1.17) |
| Week5-6 | 221797 | 85 | 38.3 | 1.03 (0.82, 1.28) | 0.62 (0.49, 0.77) |
| Dose2 |  |  |  |  |  |
| Week 1-6 | 803458 | 548 | 68.2 | 3.19 (2.85, 3.57) | 0.9 (0.8, 1.01) |
| Week1 | 133990 | 102 | 76.1 | 2.10 (1.71, 2.58) | 0.97 (0.79, 1.19) |
| Week2 | 133965 | 84 | 62.7 | 1.77 (1.42, 2.22) | 0.79 (0.63, 0.98) |
| Week3 | 133935 | 80 | 59.7 | 1.76 (1.40, 2.21) | 0.75 (0.6, 0.95) |
| Week4 | 133898 | 95 | 71.0 | 2.17 (1.75, 2.68) | 0.89 (0.72, 1.11) |
| Week5-6 | 267669 | 187 | 69.9 | 2.25 (1.92, 2.65) | 0.87 (0.74, 1.02) |
| Dose3 |  |  |  |  |  |
| Week 1-6 | 627193 | 516 | 82.3 | 3.04 (2.61, 3.53) | 0.64 (0.55, 0.75) |
| Week1 | 104603 | 69 | 66.0 | 4.01 (3.10, 5.18) | 0.58 (0.45, 0.75) |
| Week2 | 104582 | 88 | 84.1 | 5.33 (4.22, 6.72) | 0.73 (0.58, 0.92) |
| Week3 | 104556 | 101 | 96.6 | 6.49 (5.21, 8.10) | 0.84 (0.68, 1.05) |
| Week4 | 104520 | 86 | 82.3 | 5.75 (4.55, 7.28) | 0.71 (0.57, 0.9) |
| Week5-6 | 208932 | 172 | 82.3 | 6.09 (5.07, 7.31) | 0.7 (0.59, 0.84) |

*Crude model included no covariates.

†Full model included age, sex, country of birth, employed as a healthcare worker, marital status, education, COVID-19 infection, and health seeking behaviours during 2018-19 (i.e., no. of primary care visits, number of specialist outpatient visits), and prior comorbidities and treatments listed in Supplemental Table S1.
